# TriatoScore: an entomological-risk score for Chagas disease vector control-surveillance

**DOI:** 10.1186/s13071-021-04954-5

**Published:** 2021-09-25

**Authors:** Gilmar Ribeiro-Jr, Fernando Abad-Franch, Orlando M. F. de Sousa, Carlos G. S. dos Santos, Eduardo O. L. Fonseca, Roberto F. dos Santos, Gabriel M. Cunha, Cristiane M. M. de Carvalho, Renato B. Reis, Rodrigo Gurgel-Gonçalves, Mitermayer G. Reis

**Affiliations:** 1 Instituto Gonçalo Moniz, Fiocruz Bahia, Salvador, Bahia Brazil; 2 Núcleo de Medicina Tropical, Faculdade de Medicina, Universidade de Brasília, Brasília, Distrito Federal Brazil; 3 Coordenação Geral de Vigilância de Zoonoses e Doenças de Transmissão Vetorial, Secretaria de Vigilância em Saúde, Ministério da Saúde, Brasília, Distrito Federal Brazil; 4 Instituto de Tecnologias da Saúde, Centro Universitário SENAI CIMATEC, Salvador, Bahia, Brazil; 5 Laboratório Central de Saúde Pública Prof. Gonçalo Moniz, Secretaria de Saúde do Estado da Bahia, Salvador, Bahia Brazil; 6 Centro de Atenção à Saúde José Maria de Magalhães Neto, Secretaria de Saúde do Estado da Bahia, Salvador, Bahia Brazil; 7 Universidade Salvador-UNIFACS, Salvador, Bahia Brazil; 8 Laboratório de Parasitologia Médica e Biologia de Vetores, Faculdade de Medicina, Universidade de Brasília, Brasília, Distrito Federal Brazil; 9 Faculdade de Medicina da Bahia, Universidade Federal da Bahia, Salvador, Bahia Brazil; 10 Yale School of Public Health, Yale University, New Haven, CT USA

**Keywords:** Chagas disease, Triatominae, Vector control, Vector surveillance, Risk stratification

## Abstract

**Background:**

Triatomine bugs transmit Chagas disease across Latin America, where vector control-surveillance is increasingly decentralized. Locally run systems often deal with highly diverse native-vector faunas—plus, in some areas, domestic populations of non-native species. Flexible entomological-risk indicators that cover native and non-native vectors and can support local decision-making are therefore needed.

**Methods:**

We present a local-scale entomological-risk score (“TriatoScore”) that leverages and builds upon information on the ecology-behavior and distribution-biogeography of individual triatomine bug species. We illustrate our approach by calculating TriatoScores for the 417 municipalities of Bahia state, Brazil. For this, we (i) listed all triatomine bug species recorded statewide; (ii) derived a “species relevance score” reflecting whether each species is native/non-native and, if native, whether/how often it invades/colonizes dwellings; (iii) mapped each species’ presence by municipality; (iv) for native vectors, weighted presence by the proportion of municipal territory within ecoregions occupied by each species; (v) multiplied “species relevance score” × “weighted presence” to get species-specific “weighted scores”; and (vi) summed “weighted scores” across species to get municipal TriatoScores. Using standardized TriatoScores, we then grouped municipalities into high/moderate/low entomological-risk strata.

**Results:**

TriatoScores were higher in municipalities dominated by dry-to-semiarid ecoregions than in those dominated by savanna-grassland or, especially, moist-forest ecoregions. Bahia’s native triatomines can maintain high to moderate risk of vector-borne Chagas disease in 318 (76.3%) municipalities. Historical elimination of *Triatoma infestans* from 125 municipalities reduced TriatoScores by ~ 27% (range, 20–44%); eight municipalities reported *T. infestans* since Bahia was certified free of *Trypanosoma cruzi* transmission by this non-native species. Entomological-risk strata based on TriatoScores agreed well with Bahia’s official disease-risk strata, but TriatoScores suggest that the official classification likely underestimates risk in 42 municipalities. Of 152 municipalities failing to report triatomines in 2006–2019, two and 71 had TriatoScores corresponding to, respectively, high and moderate entomological risk.

**Conclusions:**

TriatoScore can help control-surveillance managers to flexibly assess and stratify the entomological risk of Chagas disease at operationally relevant scales. Integrating eco-epidemiological, demographic, socioeconomic, or operational data (on, e.g., local-scale dwelling-infestation or vector-infection frequencies, land-use change and urbanization, housing conditions, poverty, or the functioning of control-surveillance systems) is also straightforward. TriatoScore may thus become a useful addition to the triatomine bug control-surveillance toolbox.

**Graphical abstract:**

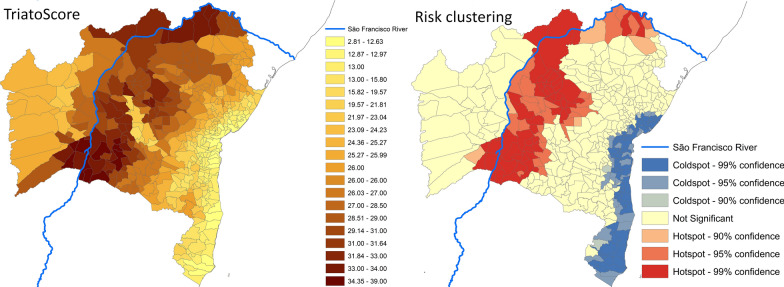

**Supplementary Information:**

The online version contains supplementary material available at 10.1186/s13071-021-04954-5.

## Background

Public health researchers, managers, policy-makers, and international agency officials make regular use of indices or indicators of population health and well-being [1, 2]. Such indices range from simple counts or rates of disease cases or events to more complex measurements of human development or socioeconomic deprivation [1–3]. Health-related indices aim primarily at summarizing population-level information on health and disease, on disease risk or exposure, or on the performance of health systems, policies or interventions in a given place (from local to global) and over a given time period (from weeks to centuries) [1, 2]. In the case of insect-borne diseases, entomological indices are essential to guide control-surveillance efforts [4–8].

Chagas disease is among the most important vector-borne diseases in the Americas; estimates for 2019 suggest that about 6.4 million people (95% uncertainty interval [UI], 5.5–7.3 million) are infected with its causal agent, *Trypanosoma cruzi*, in Latin America, leading to annual losses of ~ 274,000 disability-adjusted life years (UI, 170,000–490,000) [9–11]. *Trypanosoma cruzi* is transmitted by 140+ species of blood-sucking triatomine bugs, and vector control-surveillance is, together with blood- and organ-donor screening, the cornerstone of disease prevention [7, 8, 11, 12]. Entomological indices are critical for the planning, running, and evaluation of Chagas disease vector control-surveillance systems [7, 8]. In particular, for a given vector species, place, and time period, the World Health Organization advocates the use of indices measuring (i) vector presence, breeding, and abundance inside and/or around houses (indexed by, respectively, infestation, colonization, and density); (ii) vector infection with *T. cruzi*; and (iii) “dispersion” across localities within a given territory—an index that helps measure progress towards local elimination of non-native species (see definitions in [7, 8]).

Each of these entomological-risk indices, however, has important practical limitations. The most general setback is that, to calculate them, control managers need to regularly organize expensive area-wide, door-to-door surveys that are difficult to maintain in resource-limited settings [11, 12]. Even in the hands of highly trained staff, moreover, routine vector and parasite detection methods have imperfect sensitivity, and indices that rely on those methods will almost always be biased down [12, 13]. In particular, there is growing evidence that (i) not all vectors will be detected during routine dwelling inspections, especially after insecticide spraying reduces bug densities [13–15], and (ii) not all *T. cruzi* infections will be detected during routine vector testing [16]. To further complicate things, local control-surveillance systems frequently deal with a wide variety of native triatomine bug species—plus, at times, introduced populations of non-native species [11, 12, 17–20]. Because different triatomine bug species or populations can play very different roles in disease transmission, control-surveillance tactics must be tailored to the local vector fauna [11, 12, 19, 20]. Although working classifications of Chagas disease vectors are available for this purpose [7, 19], they often (i) lack the flexibility needed to track key time-varying events (such as, say, the local elimination of non-native species and their replacement by less-known native vectors) and (ii) tend to conflate biological and operational considerations, which makes them confusing and potentially ineffective [11, 12, 20, 21].

In the current context of decentralized health systems across Latin America, Chagas disease vector control-surveillance decisions are increasingly made at the local level [22, 23]. Flexible entomological-risk indicators that work well for both native and non-native vector species and that can support local, timely decision-making are therefore needed [20]. Here, we present an entomological-risk score (“TriatoScore”) that leverages and builds upon information on the ecology, behavior, distribution, and biogeography of individual triatomine bug species and can help control-surveillance managers to assess, stratify, map, and monitor Chagas disease entomological risk at operationally relevant scales. To illustrate the use of TriatoScore in practice, we applied our approach to the 417 municipalities of Bahia state, Brazil, where native vectors are highly diverse and Chagas disease remains a major public health concern.

## Methods

We aimed at deriving an entomological-risk score based on vector species potentially involved in Chagas disease transmission at the relatively fine scale matching the needs of decentralized control-surveillance [23]. This raised two general issues. First, we needed to allow for the sharp differences in epidemiological relevance across vector species [7, 19, 24]. To do this, we combined the hierarchical working classification of triatomine bug species, populations, foci, and individuals proposed by one of us (see [20] and Table 1) with current knowledge about the ecological-behavioral features that drive such interspecies differences [7, 11, 12, 18, 19, 24–26]. Second, we needed to derive a reliable picture of each species’ geographic distribution. Because local-scale bug presence data are almost inevitably incomplete, we complemented actual records (see below and [27–33]) with the best available knowledge about the ecoregional biogeography of each species—following [17, 34] and hence based on Dinerstein et al.’s proposal [35, 36]. We then applied this approach to the 400+ municipalities of the northeastern Brazilian state of Bahia; below we (i) briefly describe the setting and data used for this illustrative study; (ii) provide a step-by-step guide to calculating municipality-specific entomological-risk scores (“TriatoScores”); and (iii) show how TriatoScores can then be used to flexibly stratify and map Chagas disease entomological risk at the municipality scale.

**Table 1:** Triatomine bug species in Bahia state, Brazil: “species relevance score” based on the approach of Abad-Franch [20]

Species or species pair	Hierarchical level				Species relevance score
	Species	Populations	Foci	Individuals	
*Triatoma infestans*^a^	Non-native	Non-wild	Domestic/peridomestic	In foci	10
*Triatoma rubrofasciata*	Non-native	Non-wild	Domestic/peridomestic	In foci	5
*Triatoma juazeirensis/brasiliensis*^b^	Native	Wild/non-wild	Domestic/peridomestic	In foci/invaders	4
*Triatoma sordida*	Native	Wild/non-wild	Peridomestic/domestic	In foci/invaders	4
*Triatoma pseudomaculata*	Native	Wild/non-wild	Peridomestic/domestic	In foci/invaders	4
*Panstrongylus megistus*	Native	Wild/non-wild	Peridomestic/domestic	In foci/invaders	4
*Triatoma lenti/bahiensis*^b^	Native	Wild/non-wild	Peridomestic/domestic	Invaders/in foci	4
*Triatoma vitticeps*	Native	Wild/non-wild	Peridomestic/rarely domestic	Invaders/in foci	3
*Triatoma costalimai*	Native	Wild/non-wild	Peridomestic/rarely domestic	Invaders/in foci	3
*Panstrongylus lutzi*	Native	Wild/non-wild	Peridomestic/rarely domestic	Invaders/in foci	3
*Rhodnius neglectus*	Native	Wild/non-wild	Peridomestic/rarely domestic	Invaders/in foci	3
*Rhodnius nasutus*	Native	Wild/non-wild	Peridomestic/rarely domestic	Invaders/in foci	3
*Triatoma melanica*	Native	Wild/non-wild	Peridomestic/rarely domestic	Invaders/in foci	3
*Triatoma tibiamaculata*	Native	Wild	Natural	Frequent invaders	3
*Panstrongylus geniculatus*	Native	Wild	Natural	Frequent invaders	3
*Triatoma petrocchiae*	Native	Wild	Natural	Invaders	2
*Triatoma sherlocki*	Native	Wild	Natural	Invaders	2
*Triatoma melanocephala*	Native	Wild	Natural	Invaders	2
*Panstrongylus diasi*	Native	Wild	Natural	Invaders	2
*Panstrongylus lenti*	Native	Wild	Natural	Invaders	2
*Cavernicola pilosa*	Native	Wild	Natural	Rare invaders	1
*Rhodnius domesticus*	Native	Wild	Natural	Rare invaders	1
*Psammolestes tertius*	Native	Wild	Natural	Rare invaders	1
*Parabelminus yurupucu*	Native	Wild	Natural	Rare invaders	1

^a^The most dangerous domestic vector of *Trypanosoma cruzi*

^b^Merged because most records available do not distinguish the species within each pair; we note that (i) most, if not all, “*T. brasiliensis*” records from Bahia most likely refer to *T. juazeirensis*, and (ii) genetic similarity between *T. lenti* and *T. bahiensis* suggest they might be conspecific [17]

### Study setting and triatomine bug data

**Fig. 1:**
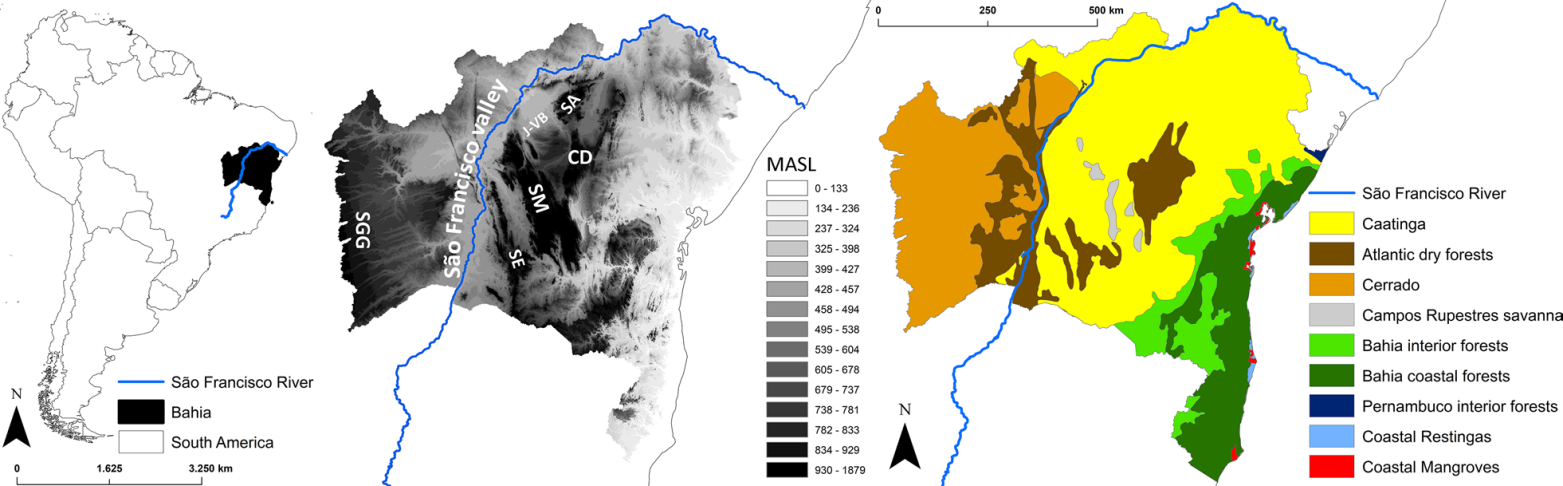
The state of Bahia, Brazil: location, relief, and ecoregions. The relief map shows altitude (meters above sea level, MASL) and major topographic features: São Francisco River valley, Serra Geral de Goiás (SGG), Serra do Espinhaço (SE), Serra da Mangabeira (SM), Chapada Diamantina (CD), Serra do Angelim (SA), and Jacaré-Verde basin (J-VB)

The state of Bahia, Brazil, has 417 municipalities in ~ 565,000 km^2^ and nearly 15 million residents, of which ~ 30% (or ~ 4.5 million people) live in rural areas [37]. By the time the last demographic census was conducted by the Brazilian Instituto Nacional de Geografia e Estatística (IBGE; 2010), about 60% of houses in the state were considered to be in poor (57%) or very poor condition (3%) [37]. Bahia’s natural vegetation cover corresponds to nine ecoregions [35, 36] that can be grouped into three major habitat classes—dry-semiarid (Caatinga and Atlantic dry forest), savanna-grassland (Cerrado and Campos Rupestres), and moist forests (Bahia coastal and interior forests, Pernambuco interior forest, and coastal Restingas and Mangroves) (see Table 2, Fig. 1 and [36]). Twenty-six triatomine bug species, of which 24 are known to be native to the state’s territory, have so far been recorded in Bahia [27–33] (Tables 1, 2). Bahia’s state health department (SESAB hereafter) uses entomological, epidemiological, demographic, socioeconomic and environmental indicators to group municipalities into three Chagas disease risk strata [32, 38, 39]; currently, 101 municipalities are considered at “low” disease risk, 214 at “moderate” risk, and 102 at “high” disease risk [38]. Recent estimates by the Global Burden of Disease (GBD) study suggest that ~ 220,000 people (UI, ~ 190,000–259,000) may carry *T. cruzi* in Bahia, with ~ 5.9–7.5 thousand new infections and ~ 300–1000 deaths attributable to Chagas disease occurring annually since 2010 [10].

**Table 2:** Triatomine bug species in Bahia state, Brazil: ecoregional biogeography and municipality-level occurrence frequency

Species or species pair	Habitat class and ecoregion^a^									Notes^b^	Municipalities
	Dry-semiarid		Savanna-grassland		Moist forest						
	Ca	ADF	Ce	CRS	BIF	BCF	PIF	Re	Ma		
*Triatoma infestans*	–	–	–	–	–	–	–	–	–	As reported after 2006/since 2000/at any time	8/31/133
*Triatoma rubrofasciata*	–	–	–	–	–	–	–	–	–	As reported	4
*Triatoma juazeirensis/brasiliensis*^c^	1	1	0	0	0	0	0	0	0	Across the state	269
*Triatoma sordida*	1	1	1	0	1	0	0	0	0	Across the state	342
*Triatoma pseudomaculata*	1	1	1	0	1	0	1	0	0	Across the state	342
*Panstrongylus megistus*	1	1	1	0	1	1	1	1	0	Across the state	417
*Triatoma lenti/bahiensis*^c^	1	1	0	1	0	0	0	0	0	Chapada Diamantina and southern São Francisco River valley	69
*Triatoma vitticeps*	0	1	1	0	1	0	0	0	0	Southern municipalities	51
*Triatoma costalimai*	0	0	1	0	0	0	0	0	0	On the Serra Geral de Goiás	9
*Panstrongylus lutzi*	1	1	0^d^	0	0	0	0	0	0	Across the state	277
*Rhodnius neglectus*	1^e^	1	1	0	0	0	0	0	0	Western and northern Bahia; some Caatinga populations	152
*Rhodnius nasutus*	1	1	0	0	0	0	0	0	0	North of the lower São Francisco River	15
*Triatoma melanica*	0	1	1	0	0	0	0	0	0	Southern São Francisco River valley	22
*Triatoma tibiamaculata*	0^d^	0^d^	0	0^d^	1	1	1	0	0	Across the state	267
*Panstrongylus geniculatus*	1	1	1	0	1	1	1	1	0	Across the state	417
*Triatoma petrocchiae*	1	0	0	0	0	0	0	0	0	North of the lower São Francisco River	15
*Triatoma sherlocki*	1	1	0	1	0	0	0	0	0	Serra da Mangabeira-Serra do Angelim (Jacaré-Verde basin)	24
*Triatoma melanocephala*	1	1	0	1	1	1	1	0	0	South and east of the São Francisco River	305
*Panstrongylus diasi*	0	1	1	0	0	0	0	0	0	Across the state	102
*Panstrongylus lenti*	1	1	0	0	0	0	0	0	0	Southern São Francisco River valley and adjacent areas	48
*Cavernicola pilosa*	1	1	1	0	1	1	1	0	0	Across the state	417
*Rhodnius domesticus*	0^d^	0	0	0	1	1	1	0	0	Across the state	206
*Psammolestes tertius*	1	1	1	0	0	0	0	0	0	Across the state	276
*Parabelminus yurupucu*	0^d^	0	0	0	1	1	1	0	0	Across the state	206

^a^A presence/absence (1/0) matrix (“–”, non-native species that only occur in human-made environments); Ca, Caatinga; ADF, Atlantic dry forest; Ce, Cerrado; CRS, Campos Rupestres savanna; BIF, Bahia interior forest; BCF, Bahia coastal forest; PIF, Pernambuco interior forest; Re, coastal Restingas; Ma, coastal Mangroves

^b^See Additional file 2: Figures S1–S24 for municipality-level species-specific occurrence maps

^c^Merged because most records available did not distinguish the species within each pair; we note that (i) most, if not all, “*T. brasiliensis*” records from Bahia most likely refer to *T. juazeirensis*, and (ii) genetic similarity between *T. lenti* and *T. bahiensis* suggest they might be conspecific [17]

^d^Presence records from these ecoregions most likely reflect lack of ecoregional map resolution, with suitable habitat present in some municipalities but not captured by the rough ecoregion-classification map we used (see Fig. 1 and [17]); we assigned a 0.001 value to the “weighted presence” of the species in those municipalities

^e^Local *R. neglectus* populations of northern-central Bahia seem to have adapted to Caatinga environments

We used two main sources of information to map triatomine bug species occurrence across Bahia’s municipalities: (i) routine control-surveillance records (1999–2019) from SESAB, and (ii) published reports providing either (a) occurrence records at the ecoregion or locality/municipality level [17, 27–34] or (b) species distribution maps based on ecological niche models [29, 31, 40–45]. Because many of the records available do not distinguish either *Triatoma juazeirensis* from *T. brasiliensis* or *T. lenti* from *T. bahiensis*, we treated the former pair as “*T. juazeirensis/brasiliensis*” and the latter pair as “*T. lenti*/*bahiensis*”. Thus, our analyses cover 24 taxa—22 individual species and two species-pairs (Tables 1, 2). For simplicity, in what follows we will use “species” to refer to these taxa. We note that (i) current knowledge suggests that “*T. brasiliensis*” records from Bahia most likely refer to *T. juazeirensis* [17, 29, 40] and (ii) genetic similarity between *T. lenti* and *T. bahiensis* suggest they might be conspecific [17].

### Calculating municipal TriatoScores: step-by-step procedure

Calculation of TriatoScores for the 417 municipalities of Bahia state involved the following six steps:

i. List all triatomine bug species recorded statewide (see above and Table 1);
ii. Derive a “species relevance score”, based on [20], reflecting whether each species (a) is native or non-native to the region under consideration (here, the state of Bahia), and, if native, (b) whether and how often it invades or colonizes human habitats. This “species relevance score”, hence, roughly measures the known epidemiological relevance of each taxon (see Table 1 and [20]);
iii. Tabulate each species’ presence/absence (coded 1/0, respectively) by municipality (see above and Additional file 1: Table S1). Our main analyses were done with the dataset including *T. infestans* records since 2006, which is when Brazil was certified free of *T. cruzi* transmission by this non-native species [12]; complementary analyses were done (a) with data on native vector species only and (b) with all historical records of *T. infestans* (Additional file 1: Table S1);
iv. For native vectors (Table 1), weight presence/absence by the extent of municipal territory within ecoregions occupied by each species (see above and Table 2). To compute these “weighted presence” values, we first used municipal [46] and ecoregion shapefiles [36] to calculate, for each municipality, the proportion of territory within each ecoregion (see Table 2 and Additional file 1: Table S2). Then, we multiplied each species’ presence (1) or absence (0) by the sum of municipality-specific ecoregion proportions. For each species, therefore, municipal “weighted presence” values range from 0 to 1 (Additional file 1: Table S3). We used ArcMap^®^ 10.5 [47] both to calculate ecoregion territorial shares and to map “weighted presence” values (see Additional file 2: Figures. S1–S24);
v. For each species and municipality, multiply “species relevance score” by “weighted presence” to get “weighted scores” (Additional file 1: Table S4); and
vi. Sum “weighted scores” across species to get municipal TriatoScores (Additional file 1: Tables S4, S5).

### Entomological-risk assessment: stratification, mapping, and spatial analysis

We standardized TriatoScores (to mean 0 and SD 1) and grouped municipalities into three strata of entomological risk: “high” risk (TriatoScores above 1 SD from the overall mean), “moderate” risk (TriatoScores within ±1 SD from the overall mean), and “low” risk (TriatoScores below 1 SD from the overall mean) (Additional file 1: Table S5). Note that, with this standardization-based procedure, entomological risk is evaluated, for each municipality, relative to the overall (average) risk across the state. Thus, if the TriatoScore of any municipality changes over time (because, say, one non-native species is eliminated—or newly recorded), then the statewide TriatoScore mean and SD will change also, potentially leading to some municipalities moving from one entomological-risk stratum to another. In other words, our approach to entomological-risk classification is fundamentally dynamic—it will highlight municipalities that are substantially above, substantially below, or close to the average risk at the time of the assessment, using updated statewide summary risk statistics (mean, SD) as the benchmark. To illustrate this, we calculated TriatoScores both including (Additional file 1: Table S5) and excluding (Additional file 1: Table S6) records of the two non-native species known to occur in Bahia—*T. infestans* and *T. rubrofasciata*. Importantly, our analyses of the latter dataset are in fact about the “baseline” entomological risk associated with native, and hence non-eliminable, vector species across the state’s municipalities (see [11, 20] and Table 1). Note also that these analyses are mathematically equivalent to assigning a zero “species relevance score” to *T. infestans* and *T. rubrofasciata*.

To assess the degree of agreement between our entomological-risk stratification based on TriatoScores and SESAB’s official disease-risk stratification [39], we used the *irrCAC* 1.0 package [48] in R 3.6.3 [49] to calculate Gwet’s AC_2_ coefficients [50]. Gwet’s AC_2_ suitably deals with ordinal data (via the *weights* = “*ordinal*” argument in *irrCAC* [48]) and is more robust to variation in rating frequency distributions than commonly used agreement coefficients such as Cohen’s κ [50, 51]. We note that comparing risk ratings derived from TriatoScore and SESAB’s criteria is somewhat “unfair”—TriatoScore uses only vector data, while SESAB also takes epidemiological, demographic, socioeconomic and environmental information into account [33, 38, 39]. The comparison, however, can be useful if it helps pinpoint municipalities where SESAB’s stratification indicates a *lower* disease risk than the (just) entomological risk suggested by TriatoScores. We also note that integrating additional sources of information into a TriatoScore-based assessment is straightforward—one just needs to add a new weight for each extra metric. To illustrate how this might work, we used housing quality data from the last demographic census (IBGE, 2010; [37]) to calculate, for each municipality, the proportion of houses in poor or very poor condition—a rough measure of dwelling vulnerability to infestation by triatomines [24, 25, 39] (see Additional file 1: Table S2). We then weighted municipal TriatoScores by this housing quality metric, standardized the resulting “TriatoScore-plus” values (to mean 0 and SD 1 as above), and grouped our study municipalities into three risk strata (defined using the ±1 SD criterion as above) (Additional file 1: Table S7).

We used ArcMap^®^ 10.5 [47] and the data in Additional file 1 to build municipality-scale maps of vector species-specific “weighted presence”, vector species richness, TriatoScore and TriatoScore-plus values, and risk strata—both disease-risk strata as per the official SESAB classification and entomological-risk strata based on TriatoScores. Finally, we used the “Hot and Cold Spot Analysis” tool of ArcMap^®^ 10.5 [47] to identify and map spatial clusters of municipalities with high (“hotspots”) and low (“coldspots”) TriatoScore values; these analyses were run with the Getis-Ord *G*_i_^*^ statistic [52] and the “contiguity—edges and corners”, “row standardization” and “false discovery rate correction” options [47].

## Results

**Fig. 2:**
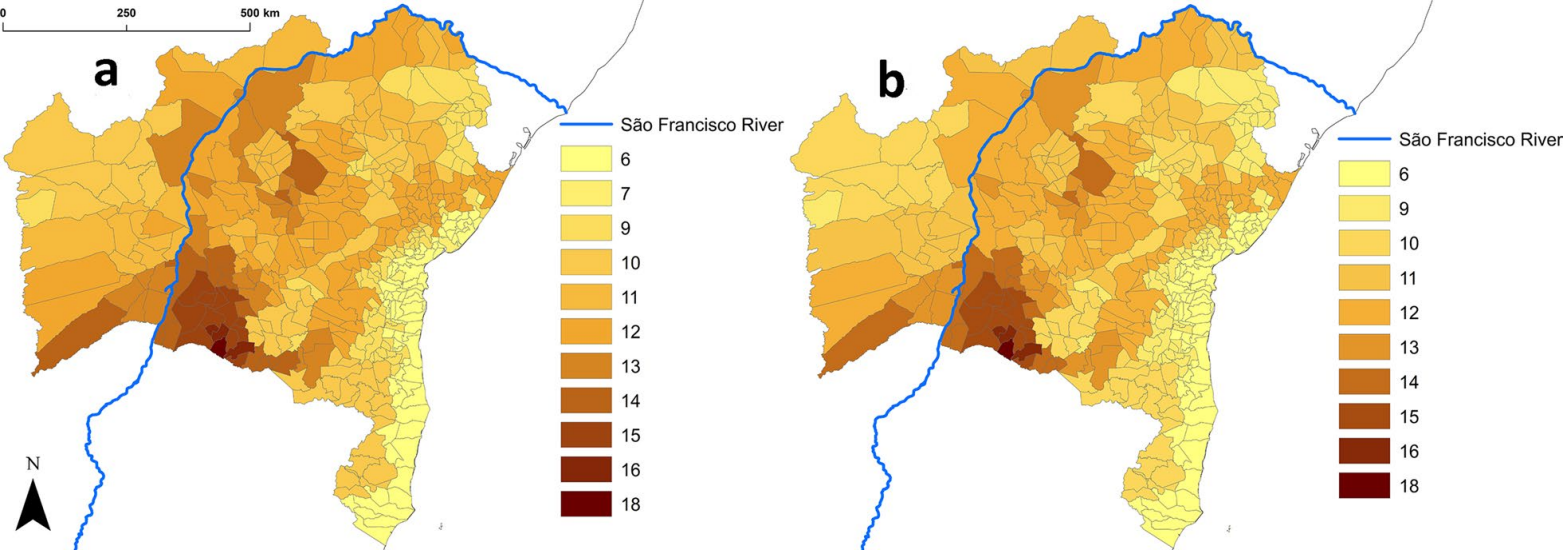
Spatial patterns of triatomine bug species richness across the 417 municipalities of Bahia state, Brazil. **a** Including records of non-native species (*Triatoma infestans* since interruption of *Trypanosoma cruzi* transmission by this species was certified in 2006 and *T. rubrofasciata*); **b** including only records of native species. Scale: number of species

Table 1 lists all triatomine bug species recorded so far in Bahia. Triatomine bugs are known or expected to occur in all of the state’s municipalities; the maps in Additional file 2: Figures S1–S24 show the municipality-level distribution of each species, with “weighted presence” values used for native species (see Additional file 1: Tables S1, S3). Our main dataset (including *T. infestans* records since 2006) reveals a mean richness of 10.2 ± 2.5 SD species (range, 6–18) per municipality (Fig. 2). Urandi is the municipality with the highest richness (18 species, all native), followed by Candiba, Jacaraci, and Pindaí (16 native species); these species-rich municipalities are located in southwestern Bahia (Fig. 2a). The geographical patterns of triatomine bug species richness do not vary substantially when only native species are considered (Fig. 2b). In Bahia, the Brazilian Atlantic dry forest is home to 17 native species, the semiarid Caatinga to 15, the Cerrado savanna to 11, and the moister Bahia interior forest to 10 species; in contrast, the coastal Mangroves (where triatomines most likely do not occur in Bahia) and Restingas (two species), as well as the higher-altitude Campos Rupestres montane savannas (three species), have the lowest richness values (Table 2). Municipality-level species richness hence seems to be somewhat higher in the ecoregion-transitional areas that roughly follow the dry San Francisco River valley and its fringes, and is clearly lower along the moister coastal region (Figs. 1, 2).

**Fig. 3:**
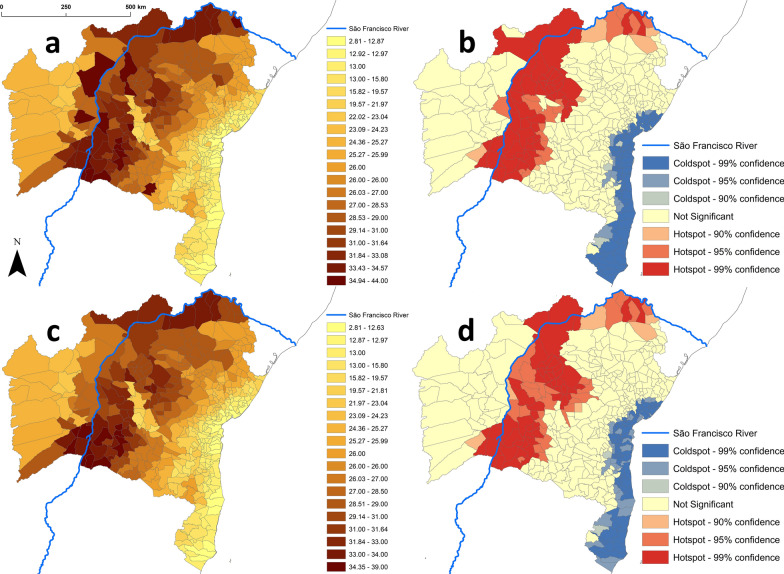
“TriatoScore”: spatial patterns of Chagas disease entomological risk across the 417 municipalities of Bahia state, Brazil. We present both raw TriatoScore values (yellow–brown scales) and the results of geospatial hotspot/coldspot analyses (red and blue scales). In **a** and **b** we used the dataset including records of both native and non-native (*Triatoma infestans* since interruption of *Trypanosoma cruzi* transmission by this species was certified in 2006 and *T. rubrofasciata*) vector species. In **c** and **d** the dataset included only records of native species

**Fig. 4:**
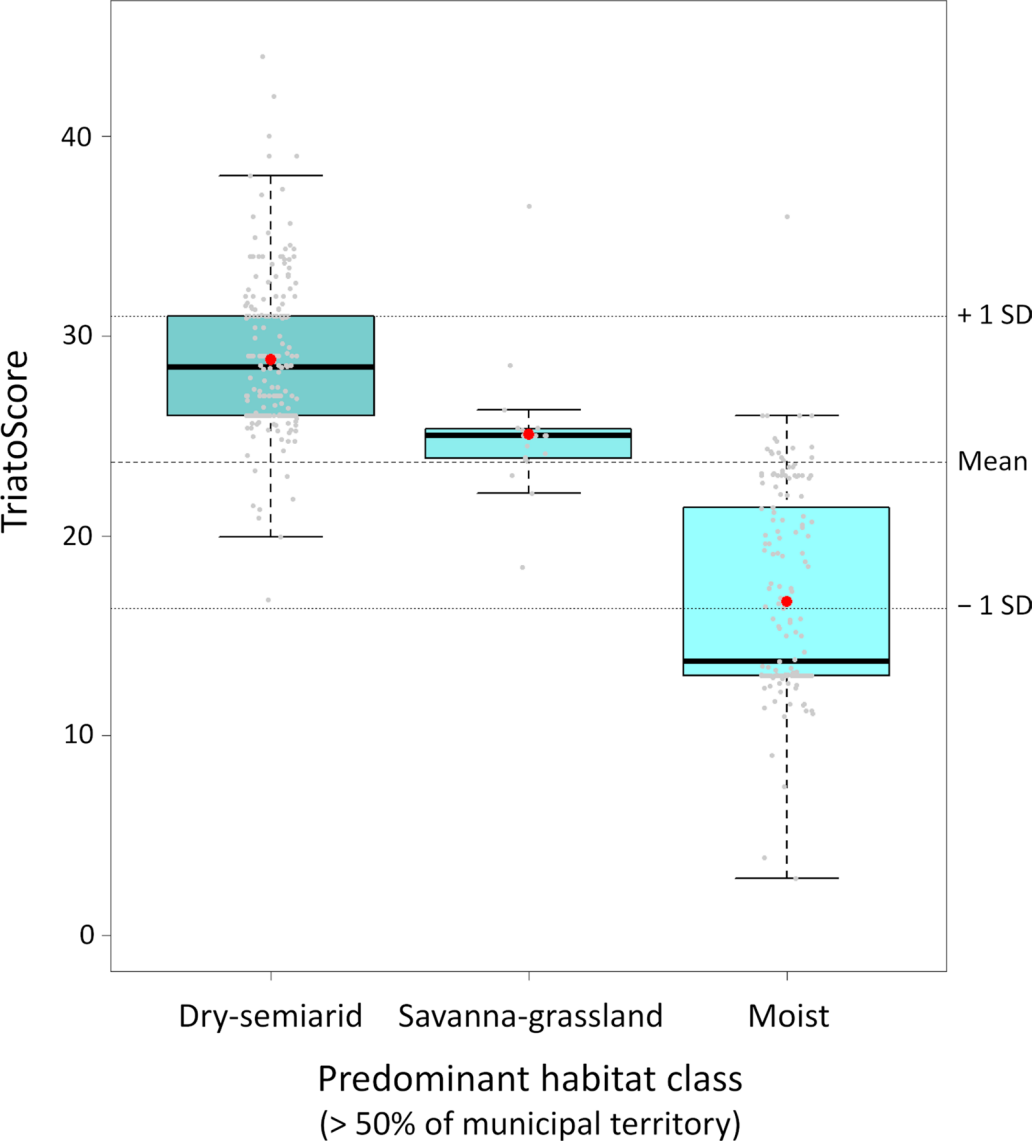
TriatoScores and predominant habitat classes across the 417 municipalities of Bahia state, Brazil. Grey dots, TriatoScore values for individual municipalities; box plots show medians (thick horizontal lines), inter-quartile ranges (box upper-lower limits), and values that fall within 1.5 times the interquartile range (whiskers); red circles are means. The dotted horizontal lines highlight the overall TriatoScore mean value and the mean ± 1 standard deviation (SD) band; in our main entomological-risk stratification scheme, we considered municipalities with TriatoScores > 1 SD above the mean as being at high risk, those with TriatoScores > 1 SD below the mean as being at low risk, and those with values within ± 1 SD of the mean as being at moderate risk

Vector species richness patterns were also reflected in municipal TriatoScore values (main dataset: mean, 23.7 ± 7.3 SD; range, 2.8–44.0; Fig. 3). TriatoScore mapping, however, revealed a clearer pattern of higher entomological risk along the São Francisco River valley and lower entomological risk along the coast; this was true both for the main dataset including non-native species (Fig. 3a) and for the native-species-only dataset (Fig. 3c). Geospatial analyses recovered a sharp hotspot cluster along the São Francisco River and the uplands that bound its valley (especially to the east and north) and a coldspot cluster along the moister coast; again, these results were largely independent of whether all vector species or only native species were considered (Fig. 3b, d). Overall, TriatoScores were higher in municipalities dominated by dry-to-semiarid ecoregions (Caatinga and Atlantic dry forest; mean TriatoScore, 28.8 ± 3.8 SD) than in those dominated by savanna-grassland (Cerrado and Campos Rupestres; mean TriatoScore, 25.1 ± 3.3 SD) or, especially, by the moist-forest ecoregions along the coast and on coastal ranges (16.7 ± 5.1 SD; Fig. 4).

**Fig. 5:**
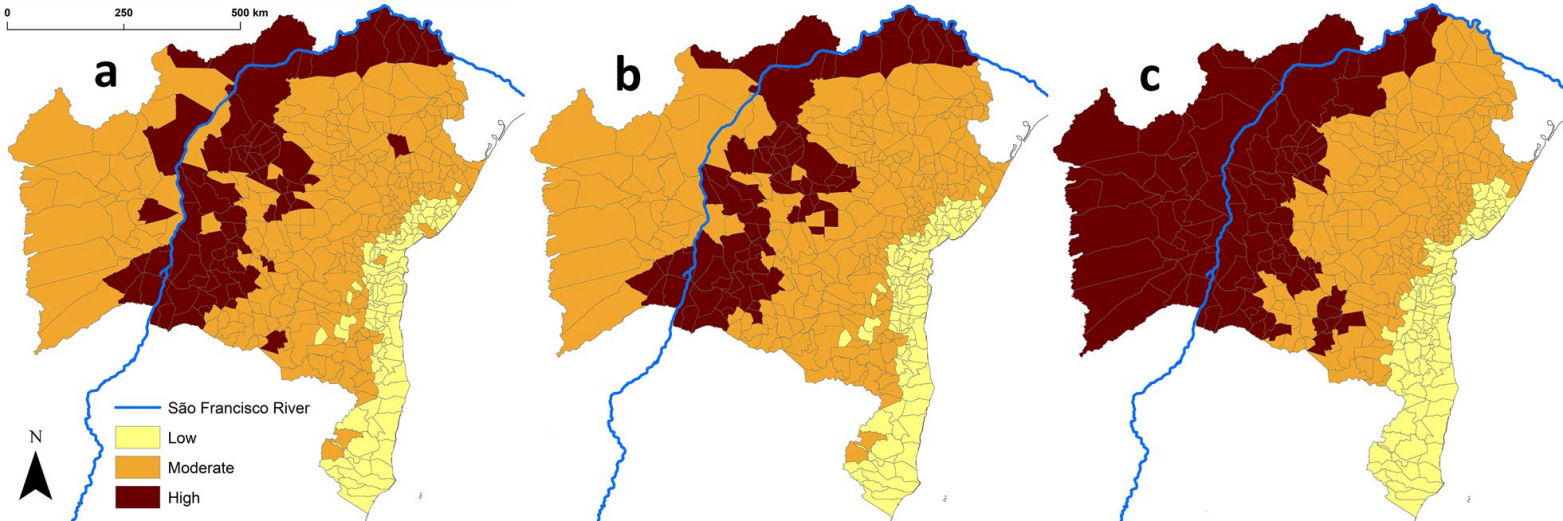
Stratification of the entomological risk of Chagas disease across the 417 municipalities of Bahia state, Brazil. **a** Risk stratification based on TriatoScore values computed with the main dataset, which includes records of non-native species (*Triatoma infestans* since interruption of *Trypanosoma cruzi* transmission by this species was certified in 2006 and *T. rubrofasciata*). **b** Risk stratification based on TriatoScore values computed with the dataset including only native species. In **a** and **b** the strata are defined as follows: high entomological risk in municipalities with TriatoScores > 1 SD above the mean, moderate risk in those with values within ±1 SD of the mean, and low risk in those with TriatoScores > 1 SD below the mean. For comparison, in **c** we map the current official disease-risk strata as defined by Bahia state’s health department; note that this stratification uses entomological, epidemiological, demographic, socioeconomic and environmental indicators, whereas TriatoScore is only based on vector data

Bahia’s SESAB official disease-risk stratification and our TriatoScore-based entomological-risk stratification agreed well (Gwet’s AC_2_ = 0.81 ± 0.02 SE), with no instances of disagreement involving high/low or low/high risk strata (Table 3). However, TriatoScore values suggest that the official classification may be underestimating risk in 42 municipalities (Table 3). Importantly, out of 152 municipalities failing to report triatomines to SESAB in 2006–2019, two (Ibiassucê and Lajedinho) had TriatoScores corresponding to high entomological risk and 71 had TriatoScores corresponding to moderate entomological risk (Additional file 1: Table S8). Figure 5 shows entomological-risk strata when TriatoScores are computed using data on all vector species (main dataset) vs. data on native species only; as summarized in Table 3, agreement with Bahia’s SESAB official disease-risk classification was again substantial (Gwet’s AC_2_ = 0.79 ± 0.02 SE). These results suggest that Bahia’s native triatomines (Tables 1, 2) can maintain high to moderate risk of vector-borne Chagas disease in 318 (76.3%) of the state’s municipalities (Fig. 5b, Table 3; Additional file 1).

**Table 3:** Agreement between the entomological-risk stratification based on TriatoScore and the official Chagas disease-risk stratification of the state’s health department (SESAB) across the 417 municipalities of Bahia state, Brazil

	SESAB (disease risk)			Total	%
	Low	Moderate	High		
All species^a^					
TriatoScore (entomological risk)					
Low	76	21	0	97	23.2
Moderate	25	176	44	245	58.8
High	0	17	58	75	18.0
Total	101	214	102	417	100
%	24.2	51.3	24.5	100	
Native species^b^					
TriatoScore (entomological risk)					
Low	78	21	0	99	23.7
Moderate	23	171	50	244	58.5
High	0	22	52	74	17.8
Total	101	214	102	417	100
%	24.2	51.3	24.5	100	

^a^Estimate of agreement, TriatoScore–all species vs. Bahia state: Gwet’s AC_2_ = 0.81 ± 0.02 SE

^b^Estimate of agreement, TriatoScore–native species vs. SESAB: Gwet’s AC_2_ = 0.79 ± 0.02 SE

**Fig. 6:**
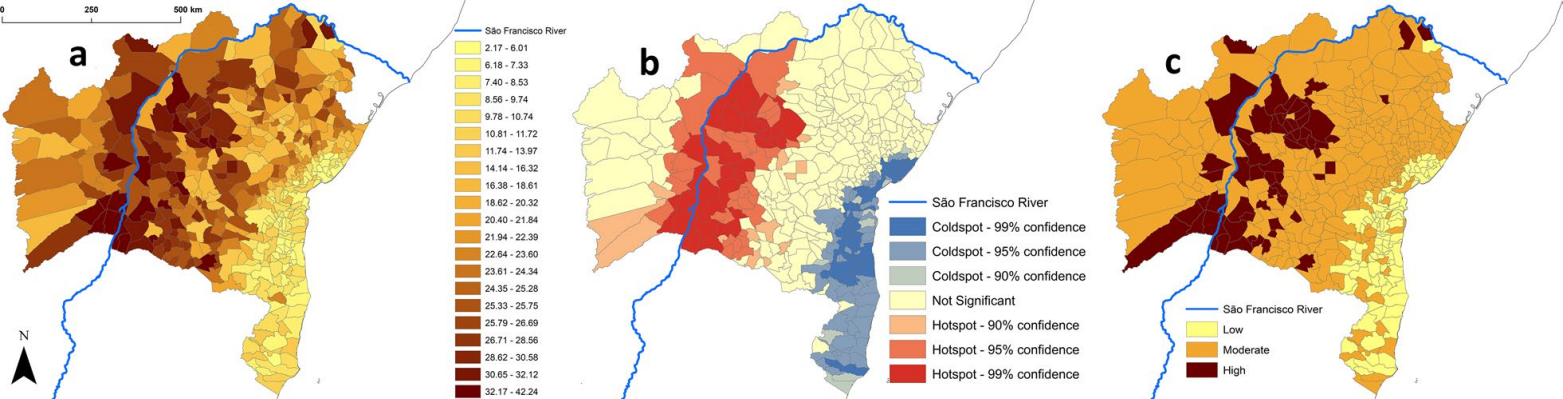
“TriatoScore-plus”: integrating housing quality data into the assessment of entomological risk. **a** Raw TriatoScore-plus values across the 417 municipalities of Bahia state, Brazil. **b** Geospatial hotspot/coldspot analysis of TriatoScore-plus values. **c** Risk strata based on TriatoScore-plus: high risk in municipalities with TriatoScores > 1 SD above the mean, moderate risk in those with values within ± 1 SD of the mean, and low risk in those with TriatoScores > 1 SD below the mean

The straightforward derivation of “TriatoScore-plus” values illustrates the potential of our approach to integrate diverse data sources—in our example, data on municipality-level housing quality. This particular extra weight reduces TriatoScores by an amount that is proportional to the share of good-quality houses in each municipality. In our case-study, reductions ranged from nil for five municipalities where that share was zero to 75.5% for two municipalities (including the state’s capital) were most houses were in good condition (see Fig. 6 and Additional file 1: Table S7). Table 4 shows the cross-classification of municipalities into risk strata based on standardized TriatoScore and TriatoScore-plus values. Note how one municipality that was at high entomological risk after TriatoScore moved to the low-risk stratum when housing conditions were considered; this reflects the fact that just 33.7% of houses were in poor or very poor condition in this municipality—Paulo Afonso, which on the other hand has a dangerous vector fauna including *T. juazeirensis/brasiliensis*, *T. sordida*, *T. pseudomaculata*, *Panstrongylus megistus*, or *P. lutzi* (Additional file 1: Tables S1, S2, S5, S7). As with TriatoScore (Table 3), TriatoScore-plus suggests that the official SESAB stratification may in some cases underestimate risk—27 and 13 municipalities that SESAB suggests are at low and moderate disease risk are classified as being at moderate and high risk, respectively, when standardized TriatoScore-plus values are used to define risk strata (Table 4). Overall, Figs. 5 and 6 show how entomological-risk patterns become less tightly associated with biogeographic-ecological features as socioeconomic information is taken into account.

**Table 4:** Agreement between risk stratification schemes based on TriatoScore-plus (which integrates housing quality data) and TriatoScore (only entomological data), and the official Chagas disease-risk classification of the state’s health department (SESAB) across the 417 municipalities of Bahia state, Brazil

	TriatoScore-plus			Total	%
	Low	Moderate	High		
TriatoScore^a^					
Low	76	21	0	97	23.3
Moderate	21	211	13	245	58.8
High	1	25	49	75	18.0
Total	98	257	62	417	100
%	23.5	61.6	14.9	100	
SESAB^b^					
Low	74	27	0	101	24.2
Moderate	24	177	13	214	51.3
High	0	53	49	102	24.5
Total	98	257	62	417	100
%	23.5	61.6	14.9	100	

^a^Estimate of agreement, TriatoScore-plus vs. TriatoScore: Gwet’s AC_2_ = 0.86 ± 0.01 SE

^b^Estimate of agreement, TriatoScore-plus vs. SESAB: Gwet’s AC_2_ = 0.79 ± 0.02 SE

## Discussion

TriatoScore is a single-figure measure of Chagas disease entomological risk that (i) covers both native and non-native triatomine bug species, (ii) tackles the issue of locally incomplete vector-occurrence records, and (iii) is designed to support decision-making at the spatial scale most relevant to decentralized control-surveillance systems. Our approach leverages the best available knowledge on the ecology-behavior and distribution-biogeography of individual triatomine bug species to compute local (e.g., municipal) TriatoScores, which can in turn be used to stratify and map entomological risk over larger spatial units. When based on standardized TriatoScores, risk stratification becomes a fundamentally dynamic exercise—changes in local vector faunas (or in our knowledge about them) are automatically accounted for as local spatial units are reassigned to risk strata relative to the recalculated average risk across all units in the region of interest. Although the most basic version of TriatoScore uses only vector data, integrating epidemiological, demographic, environmental, or operational information is straightforward. TriatoScore may hence become a useful addition to the Chagas disease vector control-surveillance toolbox.

Our approach draws primarily on the hierarchical working classification of Chagas disease vectors put forward by one of us with the suggestion that it was not only simple and biologically sound, but also potentially useful [20] see also [39]. Here, we illustrate how this idea can be put to work in practice. At the highest level of the hierarchy [20], two triatomine bug species recorded in Bahia are non-native to the state. *Triatoma infestans* is the most dangerous domestic vector of Chagas disease [7, 11, 12, 19, 20, 24, 25], and was hence given the highest “species relevance score” (Table 1). *Triatoma rubrofasciata* is strongly associated with rats of the genus *Rattus*, among which it transmits *Trypanosoma conorhini*; although it can also support *T. cruzi* infections, this originally Asian species has limited relevance as a vector of Chagas disease [17, 24–26, 53], and its “species relevance score” is therefore much lower (Table 1). Importantly, these non-native species can and should be targeted for local elimination, and this critical operational consideration sets them apart from the species that are locally native [12, 20, 39]. Native species were given “species relevance scores” ranging from 4 for those known to often breed inside and/or around houses (*T. juazeirensis/brasiliensis*, *T. pseudomaculata*, *T. sordida*, *T. lenti/bahiensis* and *P. megistus*) to 1 for those that have only rarely been found invading human dwellings (e.g., *Rhodnius domesticus* or *Parabelminus yurupucu*) (see Table 1 and [7, 11, 12, 14–17, 19–21, 24–34, 39]). While the values we chose for scoring are admittedly arbitrary, they reflect our best knowledge about the epidemiological relevance of each species—and, most importantly, the scores’ relative sizes reflect the species’ relative relevance [20]. In practice, the initial step of our approach thus entails eliciting expert opinion on the relevance of each triatomine bug species known to be present in the region of interest, with special attention paid to *relative* relevance.

The second general requirement was to map each species’ occurrence at the scale of municipalities. We used vector-presence records generated by local surveillance systems as our main data source, yet as many as 152 municipalities (36.5%) did not produce any record over the period 2006–2019—and the tally remains at 125 (or 30%) when considering the two decades since 1999 (Additional file 1: Table S8). This, of course, could not be taken to mean that triatomines do not occur (and enter houses) in any of those municipalities; rather, the absence of records almost certainly springs from the imperfect functioning of entomological surveillance [13–15, 21]. To fill in the spurious blanks in the species-by-municipality matrices and maps, we complemented this dataset with records from the literature, including (i) actual occurrence records that we could map to a municipality, (ii) species distribution maps derived from ecological niche models, and (iii) the best available knowledge about the ecoregional biogeography of each species (Table 2) [17, 27–34, 40–45].

This mapping procedure was straightforward for most species, but somewhat challenging for a few (see Table 2 and its footnotes). First, some species are local endemics restricted to a specific subarea within an ecoregion. For example, *T. sherlocki* seems to be endemic to the Jacaré-Verde basin and the ranges that bound it [17, 30–33, 40] (Fig. 1), and *T. melanica* does not seem to extend into the Cerrado to the northwest of Bahia [17, 30–33, 40, 45] (see Table 2 and Additional file 2: Figures S13, S17). Second, we found that some species occur in municipalities with supposedly unsuitable ecoregional ecologies. For example, *T. tibiamaculata*, *R. neglectus*, *R. domesticus* and *Pa. yurupucu* have been recorded in municipalities with 100% of the land classified as Caatinga (Table 2). Most such cases are readily explained by the relatively low spatial resolution of available ecoregional classifications—suitable habitat is likely present in small patches not captured by our coarse-scale ecoregion map (Fig. 1). To reflect this “marginal occurrence”, we assigned a 0.001 value to the “weighted presence” of *T. tibiamaculata*, *P. lutzi*, *R. domesticus* and *Pa. yurupucu* in municipalities with this kind of mismatch between records and ecoregions (see Table 2 and [17, 34]). The case of *R. neglectus* appears to be different—local populations of this species, which is primarily from the Cerrado [17, 24, 25, 34, 42, 43], seem to have adapted to drier Caatinga environments in northern-central Bahia [32, 33, 42] (see Table 2 and Additional file 2: Figure S11). Finally, although *P. megistus* is primarily a moist-forest species, wild populations are also common in drier ecoregions including the Cerrado and Caatinga, where they occupy gallery forests and other moister-habitat patches [17, 29]; again, our ecoregional assessment does not capture such fine-scale environmental heterogeneity. The full set of species-specific “weighted presence” maps is presented in Additional file 2: Figures S3–S24, and the data used to build them are available in Additional file 1: Tables S1–S7.

TriatoScore values were overall higher in municipalities dominated by dry-to-semiarid ecoregions, lower in municipalities where moister forests dominate, and intermediate in municipalities dominated by seasonally dry savanna-grassland (Fig. 4). The higher entomological risk in the Caatinga and Atlantic dry forest reflects both a particularly high triatomine species richness (Table 2) and the fact that many of those species are often found infesting or invading houses (Table 1) [7, 11, 12, 15, 17, 19, 24–34, 40–45]. While the eastern portion of the Cerrado that covers western Bahia is also fairly species-rich (Table 2), at least three of the 11 triatomine bug species occurring there (*Panstrongylus diasi*, *Cavernicola pilosa* and *Psammolestes tertius*) are seldom found in or around houses (Table 1) [7, 11, 12, 17, 24–34, 40–45]. In Bahia, the species-rich Caatinga, Atlantic dry forest and Cerrado meet along the São Francisco River valley (Fig. 1), and TriatoScore mapping revealed a pattern of higher entomological risk in that region (Fig. 3). In contrast, the Campos Rupestres montane savannas of the Serra da Mangabeira (Fig. 1) are home to just three triatomine bug species (Table 2) [17, 27–33], and TriatoScore values were accordingly low in ten municipalities (with ~ 10% to ~ 50% of territory corresponding to Campos Rupestres) located along a southeast-northwest diagonal, narrow strip in the center of the state (Figs. 1, 3). Finally, 6 of the 10 triatomine bug species known to occur in the moister coastal ecoregions do not seem able to stably infest houses (Tables 1, 2) [7, 11, 12, 17, 24–34]. Therefore, TriatoScore values are particularly low along the central and southern coast of Bahia (Fig. 3). Geospatial analyses confirmed these patterns by showing (i) a clear-cut hotspot of higher entomological risk in municipalities along the São Francisco valley and on the ranges that bound it (particularly to the east and north), (ii) a clear-cut coldspot of lower entomological risk in municipalities along the central-southern coast, and (iii) two separate areas of nonsignificant clustering of TriatoScore values: (a) the Cerrado-dominated western region and (b) the Caatinga-dominated region east of the central uplands (the Serra do Angelim-Chapada Diamantina-Serra da Mangabeira-Serra do Espinhaço complex) plus the overall drier northern coast (Figs. 1, 3).

One particularly attractive feature of the TriatoScore approach is that it is fundamentally dynamic. This can be illustrated with a hypothetical example. Suppose that in some year in the near future (i) *T. infestans* infestation foci are discovered in two municipalities where the species was historically present but from where it was not reported since 2006 (say, Abaré and Anagé), and (ii) intensive but negative searches strongly suggest that *T. rubrofasciata* is no longer present in two municipalities where it was recorded in the past (say, Glória and Jussiape). In Additional file 1: Table S9 we show how these changes can swiftly be incorporated into an exercise of entomological-risk assessment and stratification—by typing the new “species relevance score” values into the appropriate cells (here, “10.0” in cells B4 and B15, and “0.0” in cells C141 and C224; highlighted in red font in Additional file 1: Table S9), TriatoScores are automatically updated and standardized and risk strata automatically recalculated (see columns Z to AC in Additional file 1: Table S9). To provide a real-life (if retrospective) example of TriatoScore’s flexibility, we examined how the historical elimination of non-native *T. infestans* from 125 municipalities [27–33] changed entomological-risk patterns across Bahia. We found that TriatoScores were reduced by an average of 26.5% (range, 20.4–43.5%) in those municipalities; declines were steeper in municipalities where the “baseline” entomological risk brought about by native vectors is lower (see Additional file 1: Table S10). A further example (this time prospective) is our evaluation of “baseline” risk—what would be the patterns of Chagas disease entomological risk if the two non-native vector species still found in the state were finally eliminated (Figs. 2, 3; Additional file 1: Tables S3, S6, S10). In general, thus, our approach can swiftly incorporate new data on local-scale vector-species distribution. Note also that to quickly identify municipalities where risk is *much* higher or *much* lower than average one just needs to tinker with the threshold used to define risk strata. For example, a ±1.5 SD criterion highlights 14 municipalities with very high, and 20 with very low, entomological risk (Additional file 1: Table S11).

Finally, we again draw attention to the fact that the basic version of TriatoScore we have presented uses only vector data, yet Chagas disease transmission risk depends on a constellation of social, cultural, economic, demographic, ecological, environmental, political, and operational determinants [7, 23, 24, 39, 54–56]. For example, and as Carlos Chagas vividly described in his 1909 paper [57], poor-quality housing sets the stage for frequent contact between vectors and humans [58]. It would therefore be of interest to develop a more elaborate version of TriatoScore in which metrics describing further potential determinants of transmission could be incorporated (see, e.g., [39]). As we have shown with our computation of municipal “TriatoScore-plus” values, this is straightforward enough—all that is needed is a set of weights, one for each putative determinant, derived from measurements taken at the scale of interest (Fig. 6). We chose to illustrate this procedure with a municipality-level measure of housing conditions, but many other possibilities could merit consideration [39]. A non-exhaustive list of examples might include local-scale measures of (i) the frequencies of dwelling infestation with triatomines or of *T. cruzi* infection in humans or vectors; (ii) the levels of poverty or human development; (iii) the patterns and dynamics of land-use change and deforestation; (iv) the demographics of urban/rural populations; or (v) whether control-surveillance systems are adequately funded, staffed, and operated in each municipality.

## Conclusions

Chagas disease is caused by a multi-host zoonotic pathogen transmitted by over 100 locally native vector species, many of which persistently invade and infest houses from the USA to Argentina [7, 11, 12, 17, 19]. Completely interrupting *T. cruzi* transmission is therefore unfeasible [11, 12]. Instead, decades of hard-won experience clearly indicate that disease control, defined as the “reduction of […] incidence, prevalence, morbidity and/or mortality to a locally acceptable level” [59], is the most that we can aim for in the Americas [11]. Long-term vector control-surveillance has a major role to play in disease control [11, 12, 14], and entomological-risk stratification and mapping is and will remain a key tool for local program managers [11, 12, 20]. TriatoScore provides a sound, dynamic, and flexible means to this end—and one that, moreover, can be easily extended to integrate operationally relevant information beyond vector data.

## Supplementary Information

**Additional file 1: Table S1.** Vector presence and species richness (columns AB, AC). **Table S2.** Ecoregions and housing quality. **Table S3.** “Weighted presence” (native vectors). **Table S4.** “Weighted scores” and “TriatoScores” (column Z). **Table S5.** “TriatoScores” and risk strata (with *Triatoma infestans* since 2006). **Table S6.** “TriatoScores” and risk strata (native species only). **Table S7.** “TriatoScore-plus” values and risk strata. **Table S8.** Municipalities with no vector records from surveillance (red font, 1999–2019; black font, 2006–2019). **Table S9.** Hypothetical scenario: “weighted scores” and “TriatoScores” (column Z). **Table S10.** “TriatoScore” changes with the elimination of *Triatoma infestans*. **Table S11.** “TriatoScores” and extreme-risk strata (± 1.5 SD criterion) (with *T. infestans* since 2006). Triatomine bug species occurrence based on routine control-surveillance records (SESAB, 1999–2019) and published reports [17, 27–29, 29–31, 31–34, 40–45].

**Additional file 2: Figure S1.***Triatoma infestans* in Bahia, Brazil (since 2006). **Figure S2.** *Triatoma rubrofasciata* in Bahia, Brazil (as reported). **Figure S3.** *Triatoma juazeirensis*/*brasiliensis* in Bahia, Brazil (“weighted presence”). **Figure S4.** *Triatoma sordida* in Bahia, Brazil (“weighted presence”). **Figure S5.** *Triatoma pseudomaculata* in Bahia, Brazil (“weighted presence”). **Figure S6.** *Panstrongylus megistus* in Bahia, Brazil (“weighted presence”). **Figure S7.** *Triatoma lenti/bahiensis* in Bahia, Brazil (“weighted presence”). **Figure S8.** *Triatoma vitticeps* in Bahia, Brazil (“weighted presence”). **Figure S9.** *Triatoma costalimai* in Bahia, Brazil (“weighted presence”). **Figure S10.** *Panstrongylus lutzi* in Bahia, Brazil (“weighted presence”). **Figure S11.** *Rhodnius neglectus* in Bahia, Brazil (“weighted presence”). **Figure S12.** *Rhodnius nasutus* in Bahia, Brazil (“weighted presence”). **Figure S13.** *Triatoma melanica* in Bahia, Brazil (“weighted presence”). **Figure S14.** *Triatoma tibiamaculata* in Bahia, Brazil (“weighted presence”). **Figure S15.** *Panstrongylus geniculatus* in Bahia, Brazil (“weighted presence”). **Figure S16.** *Triatoma petrocchiae* in Bahia, Brazil (“weighted presence”). **Figure S17.** *Triatoma sherlocki* in Bahia, Brazil (“weighted presence”). **Figure S18.** *Triatoma melanocephala* in Bahia, Brazil (“weighted presence”). **Figure S19.** *Panstrongylus diasi* in Bahia, Brazil (“weighted presence”). **Figure S20.** *Panstrongylus lenti* in Bahia, Brazil (“weighted presence”). **Figure S21.** *Cavernicola pilosa* in Bahia, Brazil (“weighted presence”). **Figure S22.** *Rhodnius domesticus* in Bahia, Brazil (“weighted presence”). **Figure S23.** *Psammolestes tertius* in Bahia, Brazil (“weighted presence”). **Figure S24.** *Parabelminus yurupucu* in Bahia, Brazil (“weighted presence”). Triatomine bug species occurrence based on routine control-surveillance records (SESAB, 1999–2019) and published reports [17, 27–29, 29–31, 31–34, 40–45].

## Data Availability

All data are available in the article and its Additional files.

## Abbreviation

SESAB: Bahia’s state health department
