# Supplementary material for: TriatoScore: an entomological-risk score for Chagas disease vector control-surveillance
Source: Parasit Vectors. 2021 Sep 25;14:492. doi: 10.1186/s13071-021-04954-5 (PMC8465766; doi:10.1186/s13071-021-04954-5)

Fig S1. ***Triatoma infestans*** **(since 2006)**

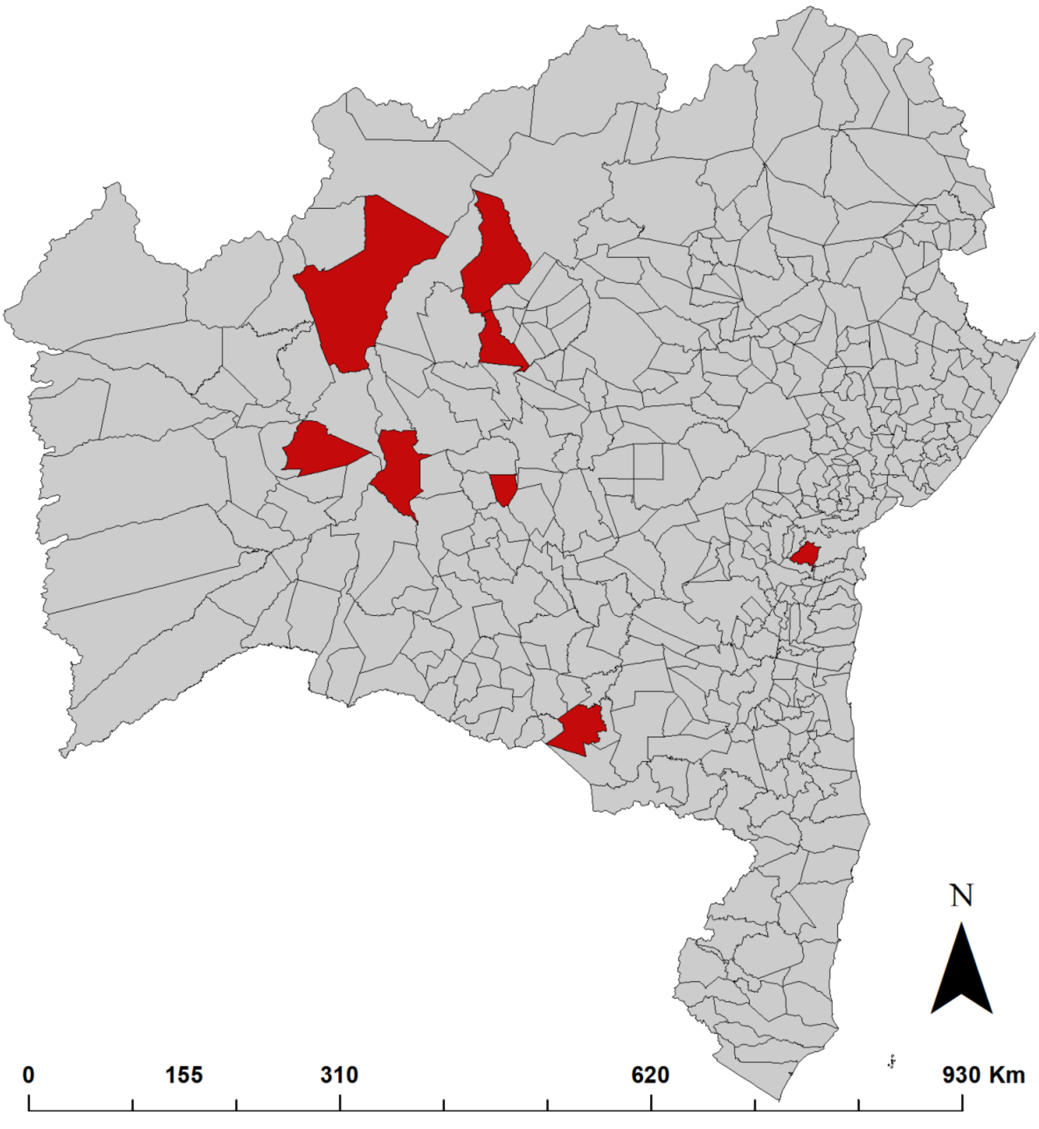

Fig S2. *Triatoma rubrofasciata*

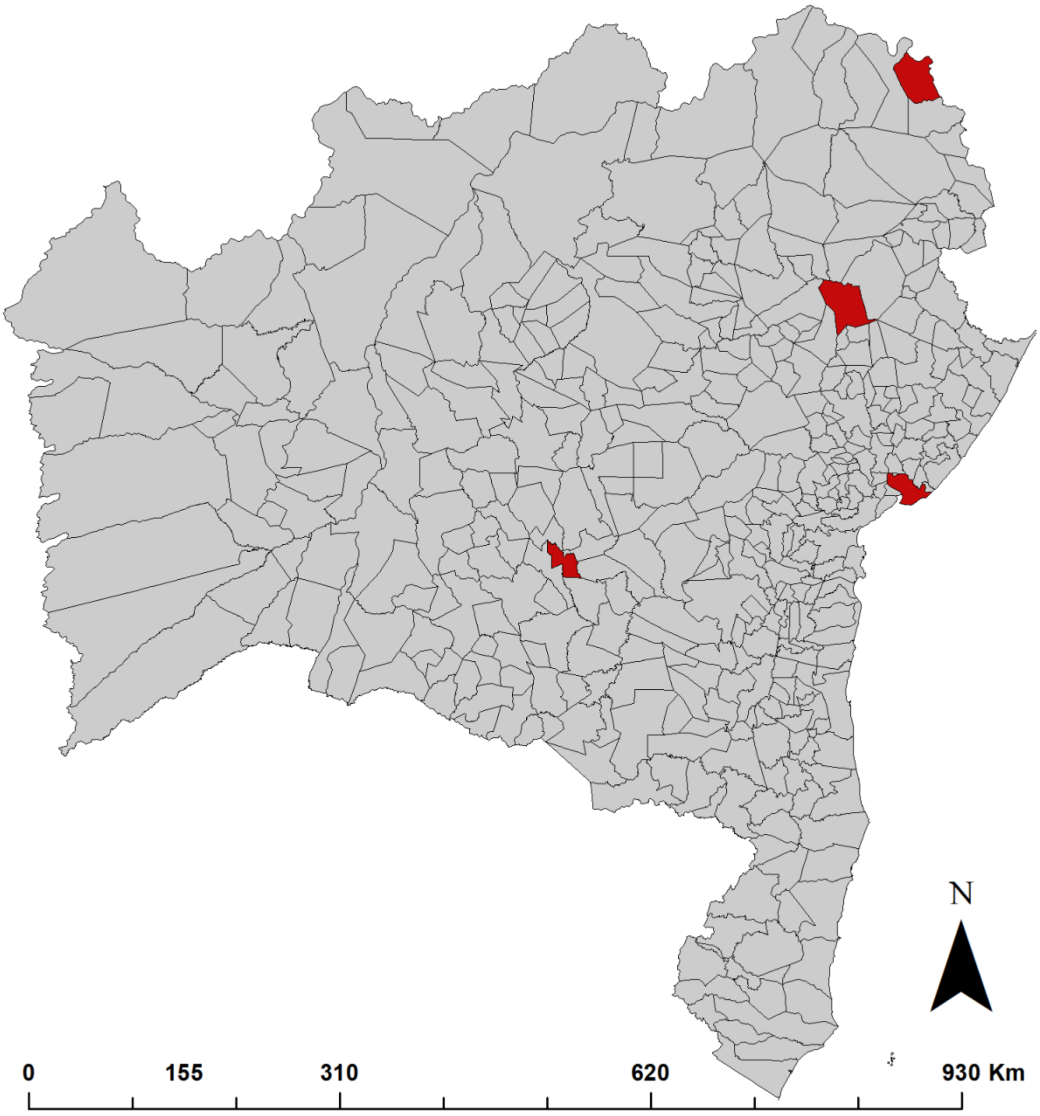

Fig S3. ***Triatoma brasiliensis/juazeirensis*** **("weighted presence")**

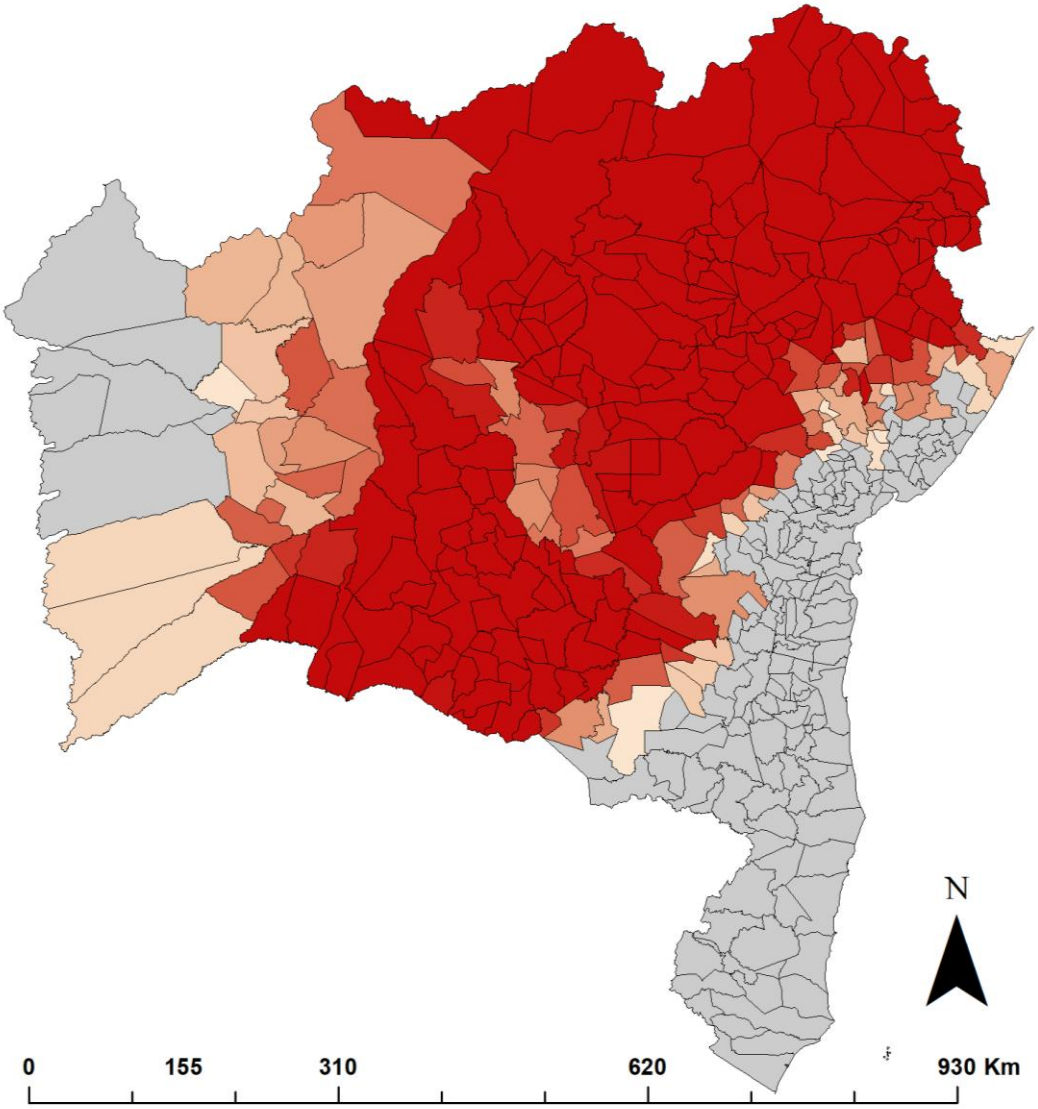

Fig S4. ***Triatoma sordida*** **(“weighted presence”)**

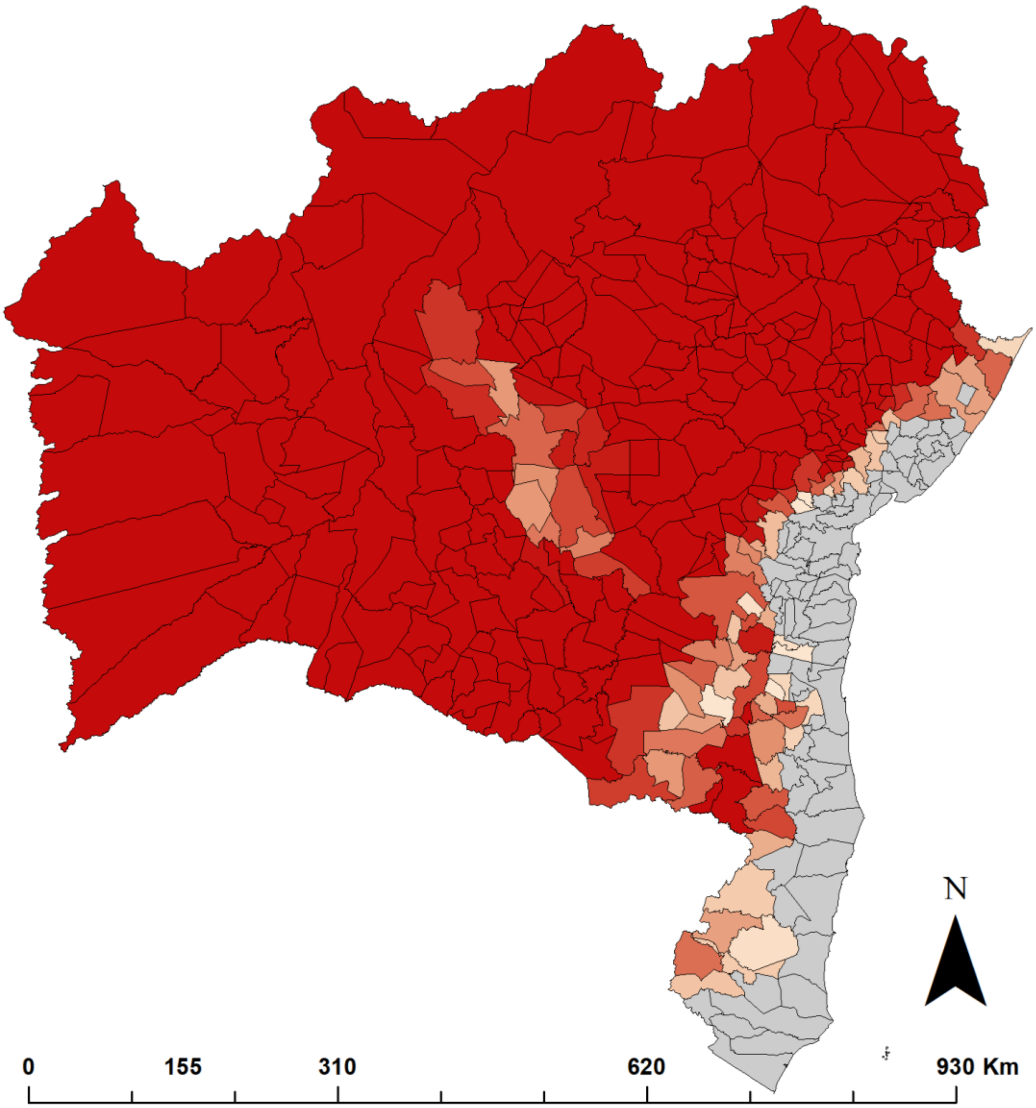

Fig S5. *Triatoma pseudomaculata* (“weighted presence”)

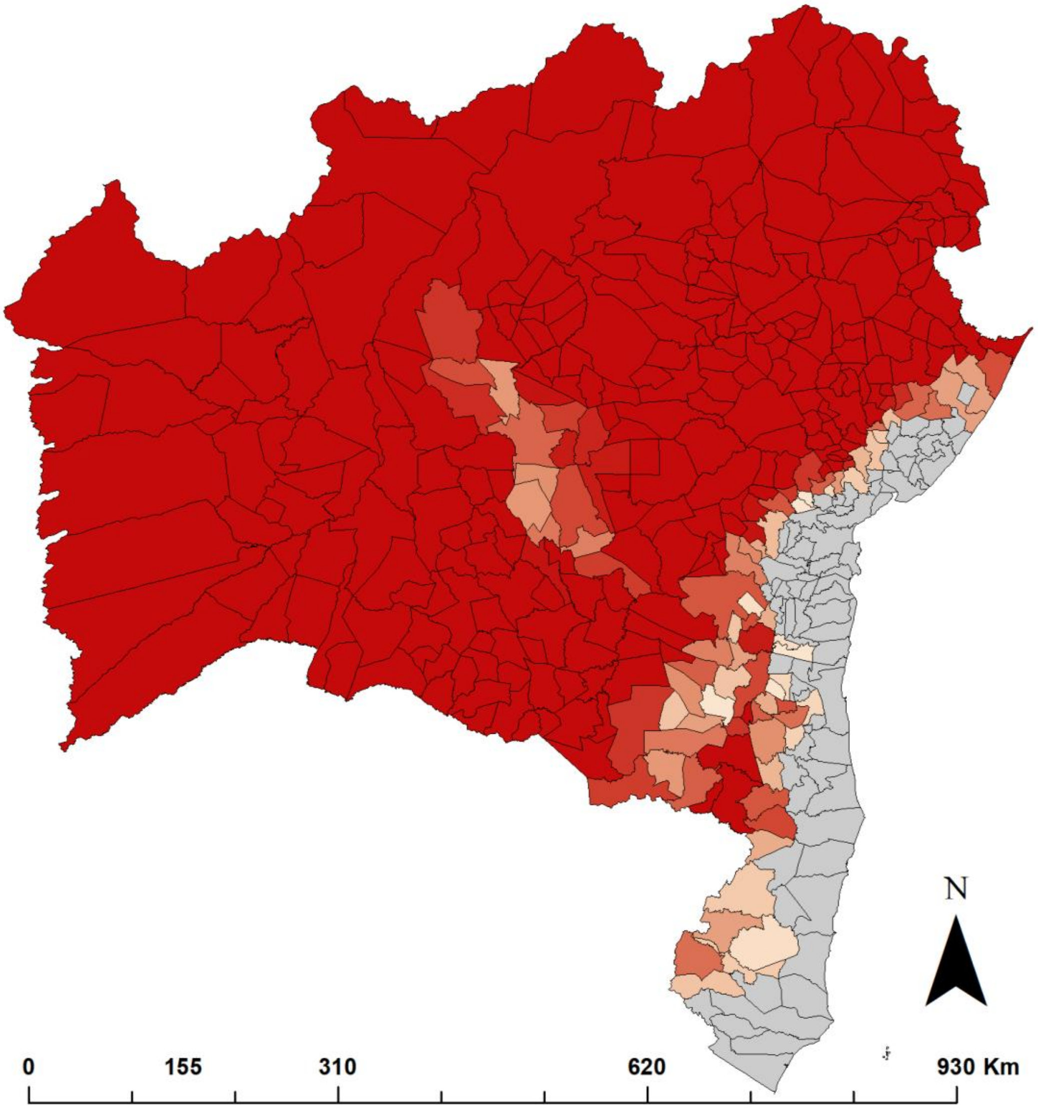

Fig S6. ***Panstrongylus megistus*** **("weighted presence")**

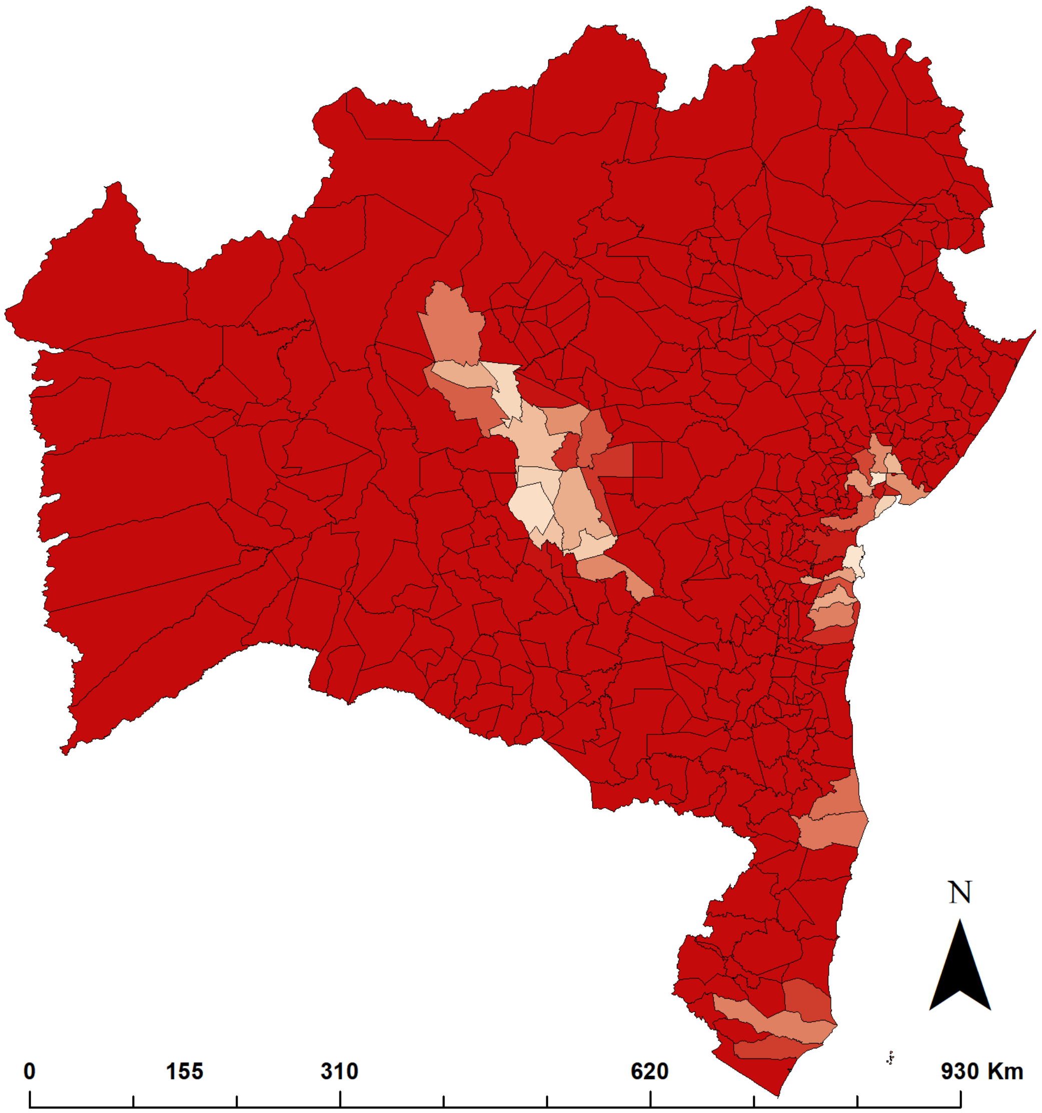

Fig S7. ***Triatoma lenti/bahiensis*** **("weighted presence")**

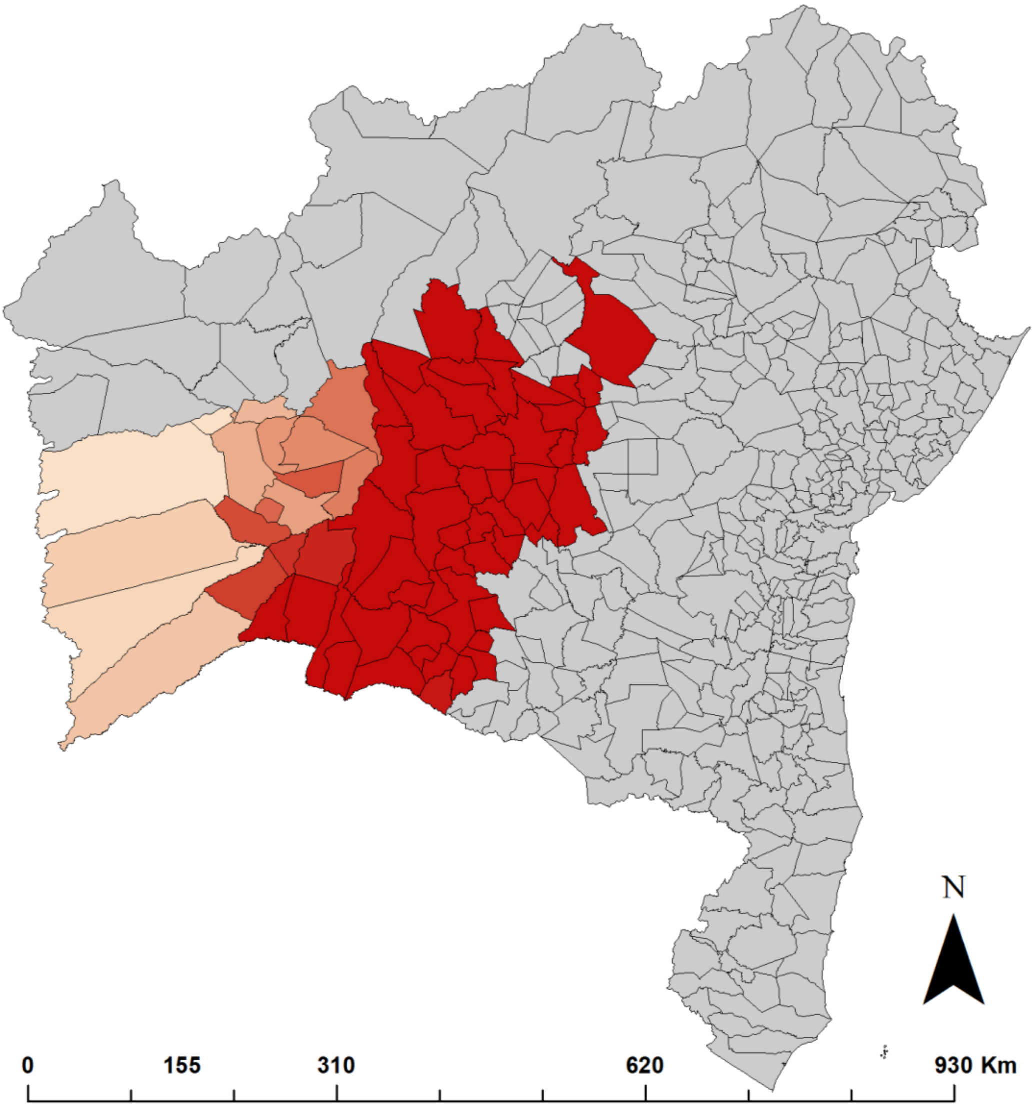

Fig S8. ***Triatoma vitticeps*** **("weighted presence")**

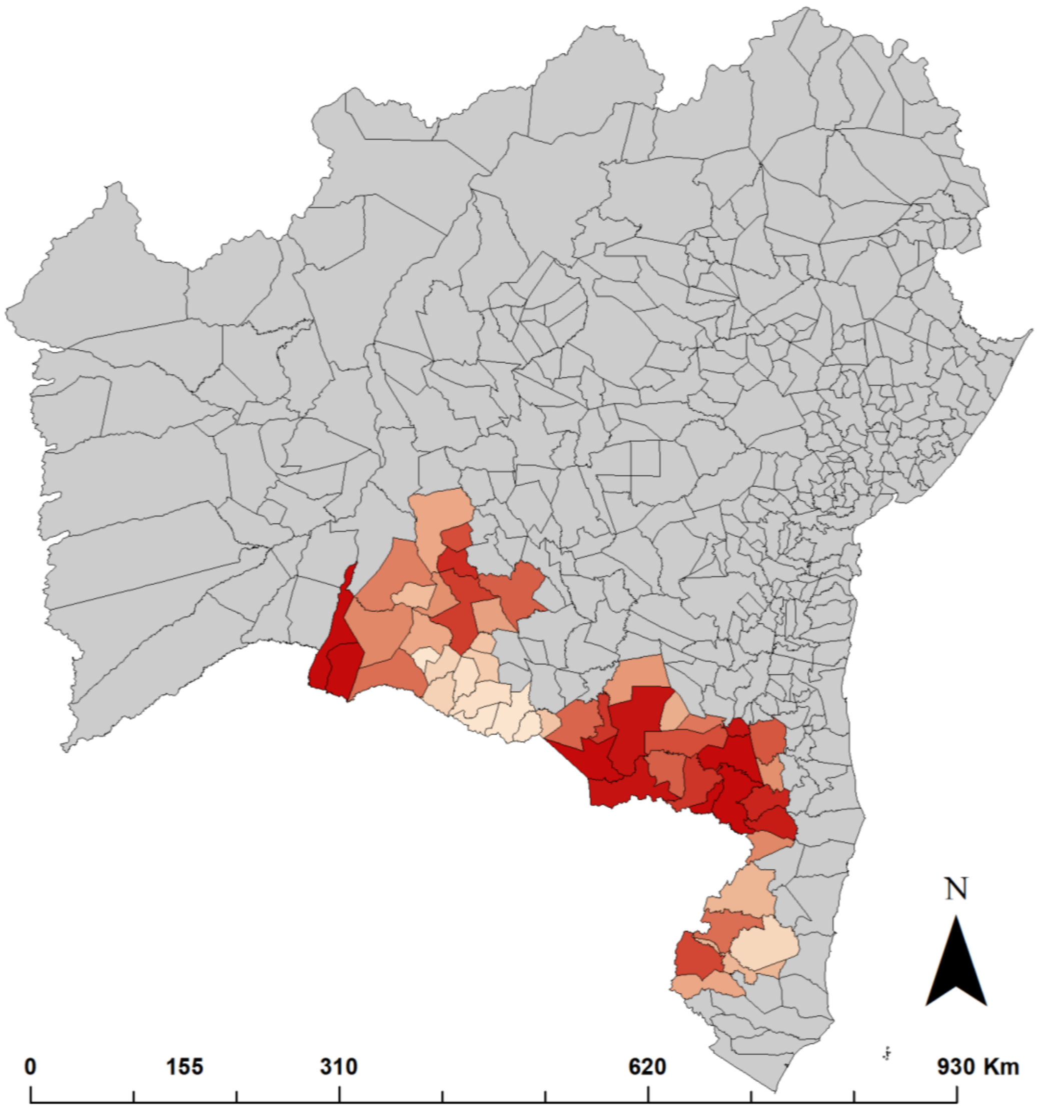

Fig S9. ***Triatoma costalimai*** **("weighted presence")**

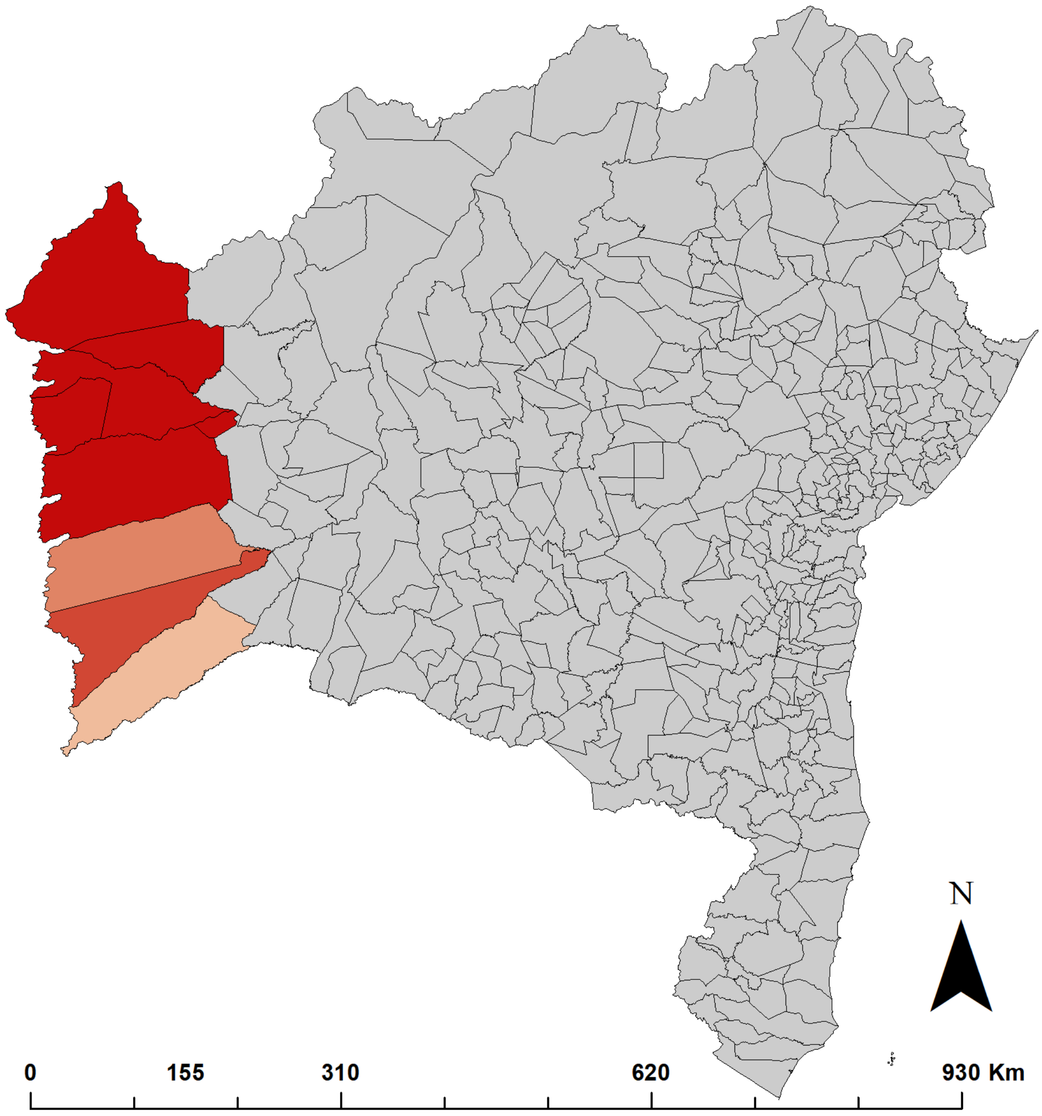

Fig S10. ***Panstrongylus lutzi*** **("weighted presence")**

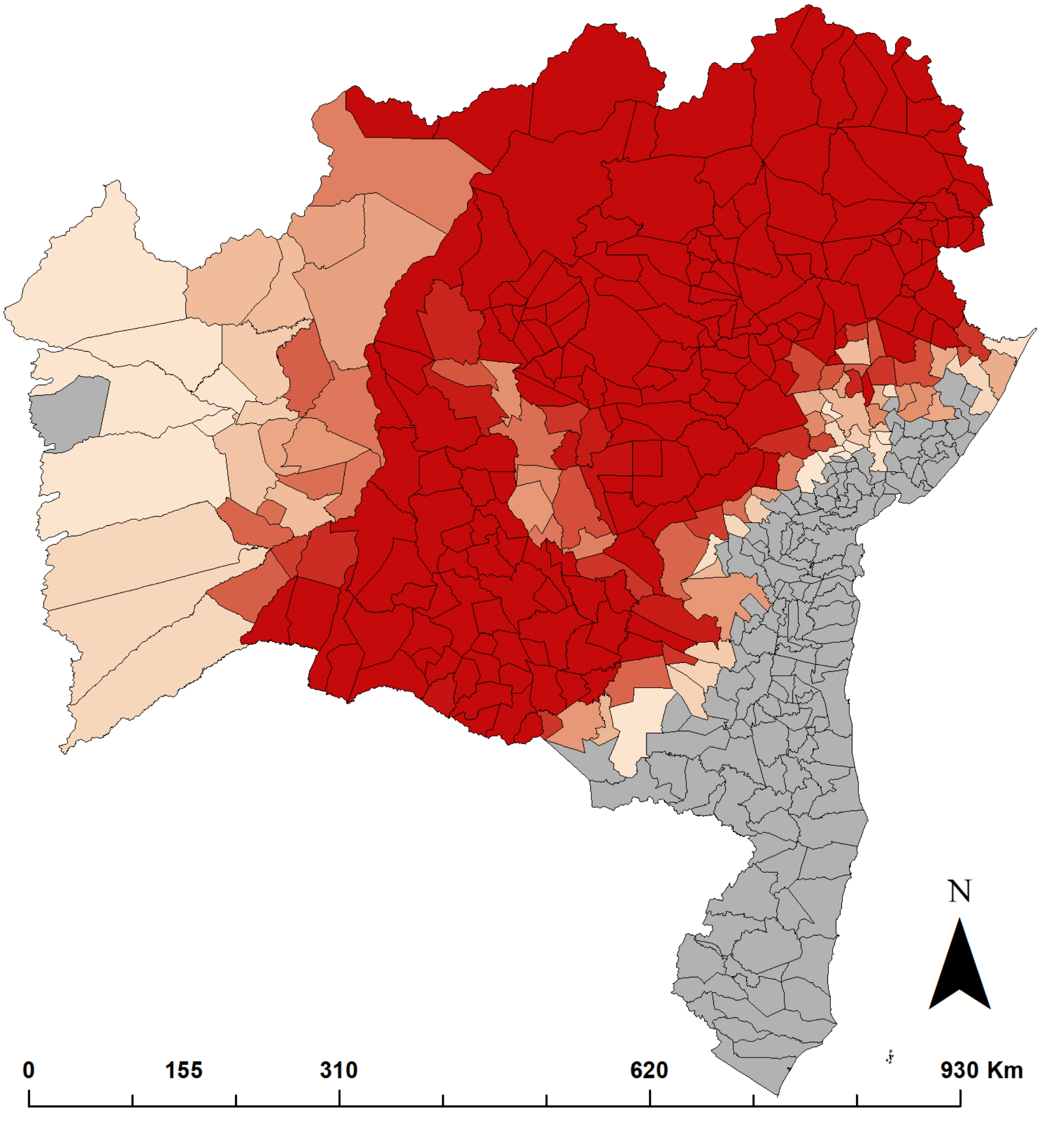

Fig S11. ***Rhodnius neglectus*** **(“weighted presence”)**

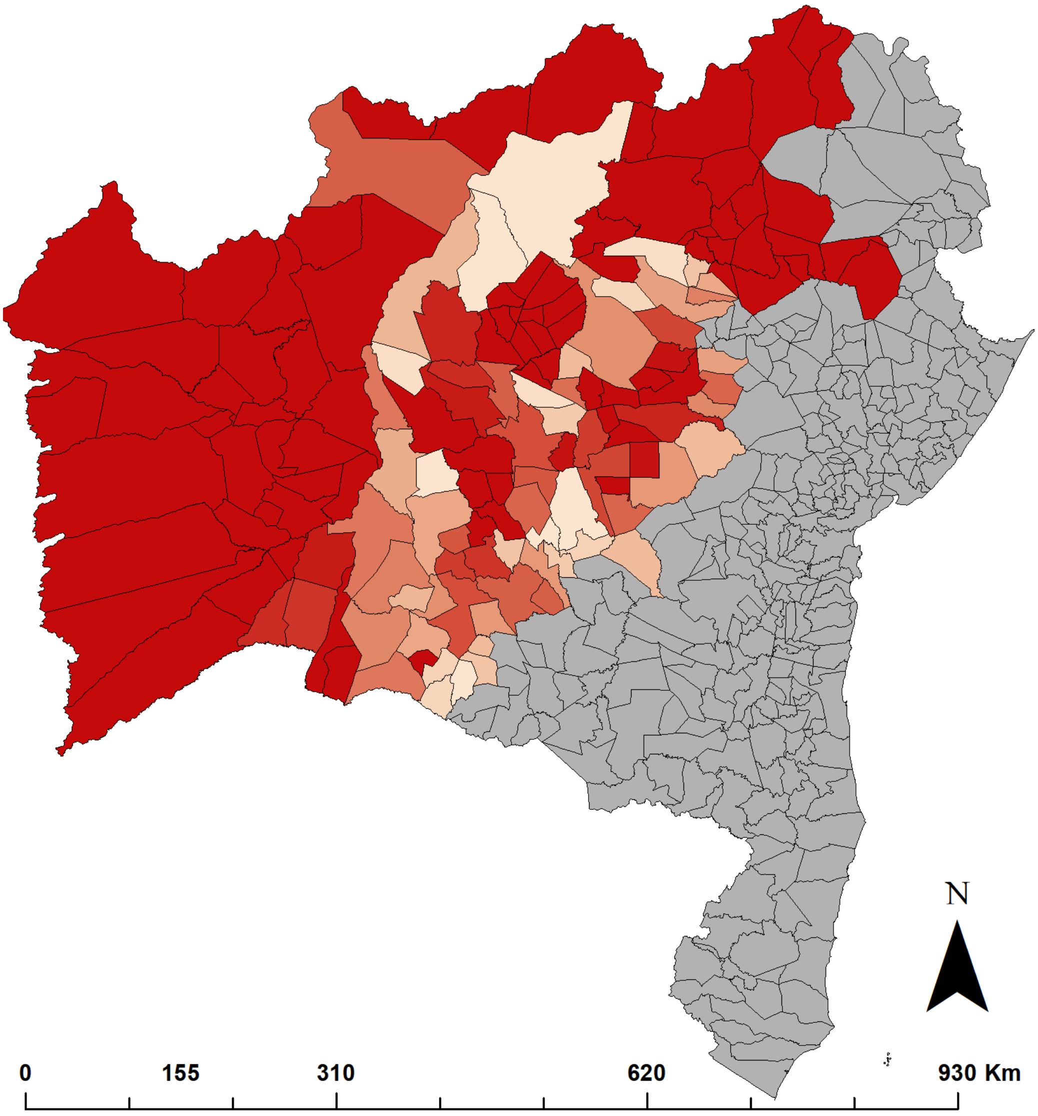

Fig S12. ***Rhodnius nasutus*** **("weighted presence")**

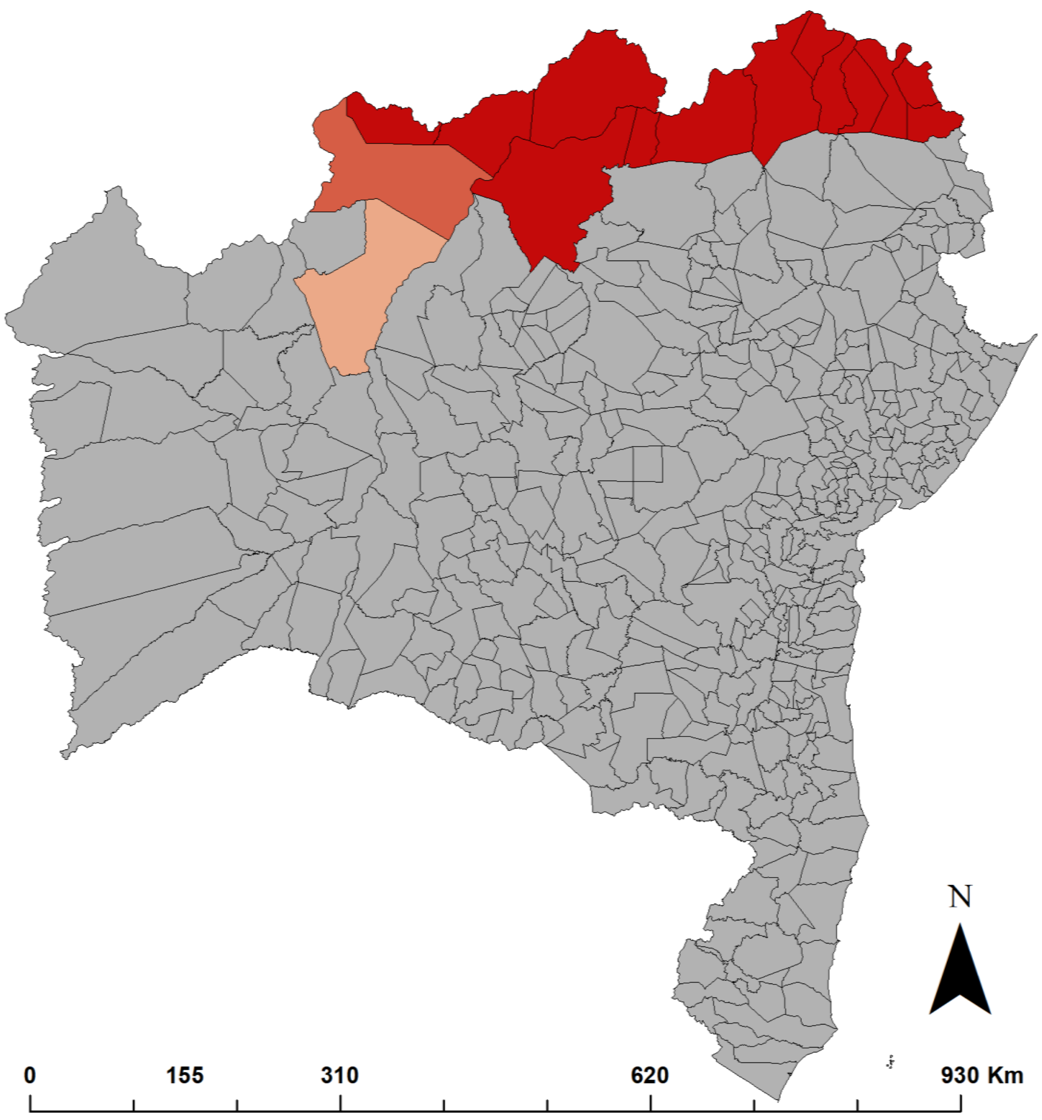

Fig S13. ***Triatoma melanica*** **("weighted presence")**

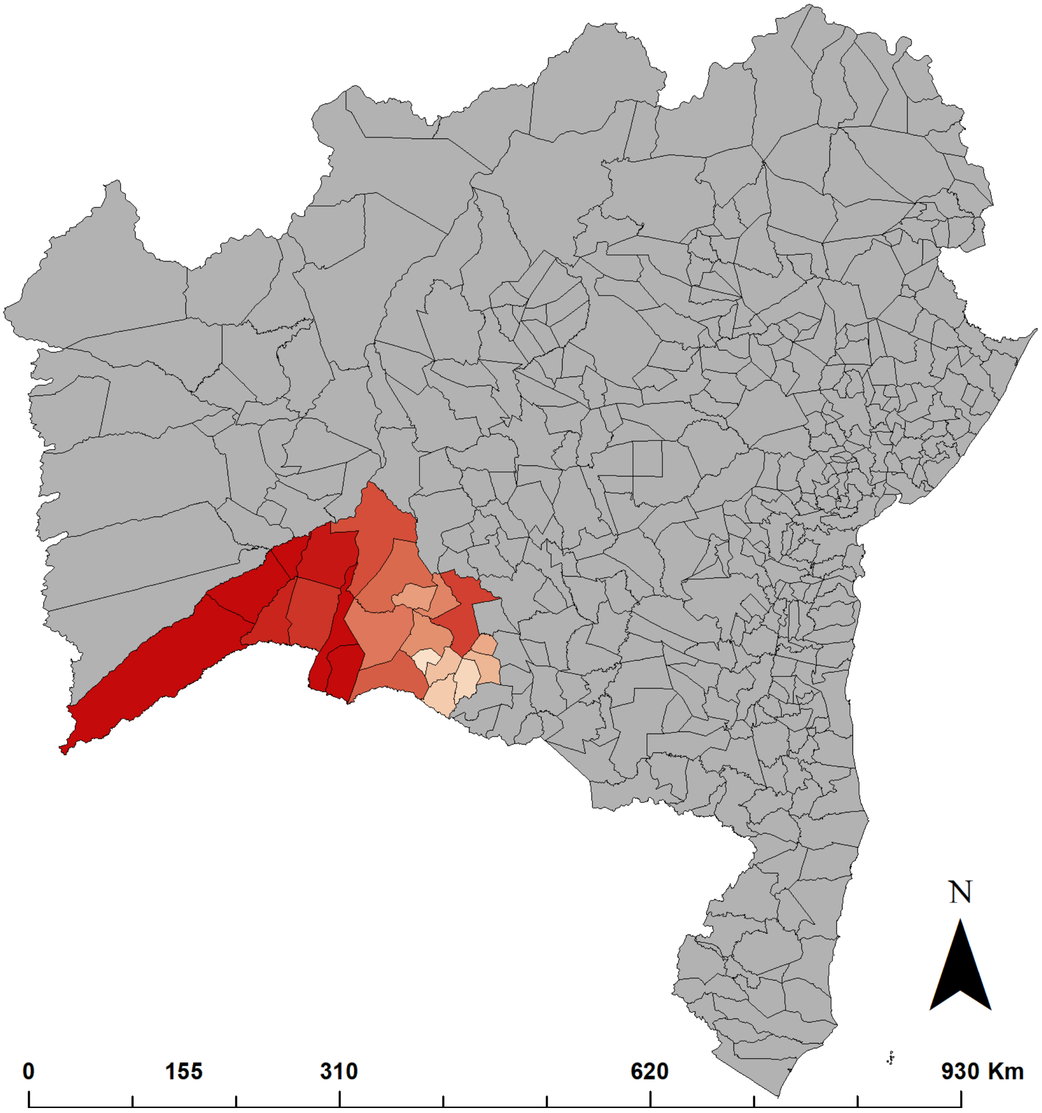

Fig S14. *Triatoma tibiamaculata* ("weighted presence")

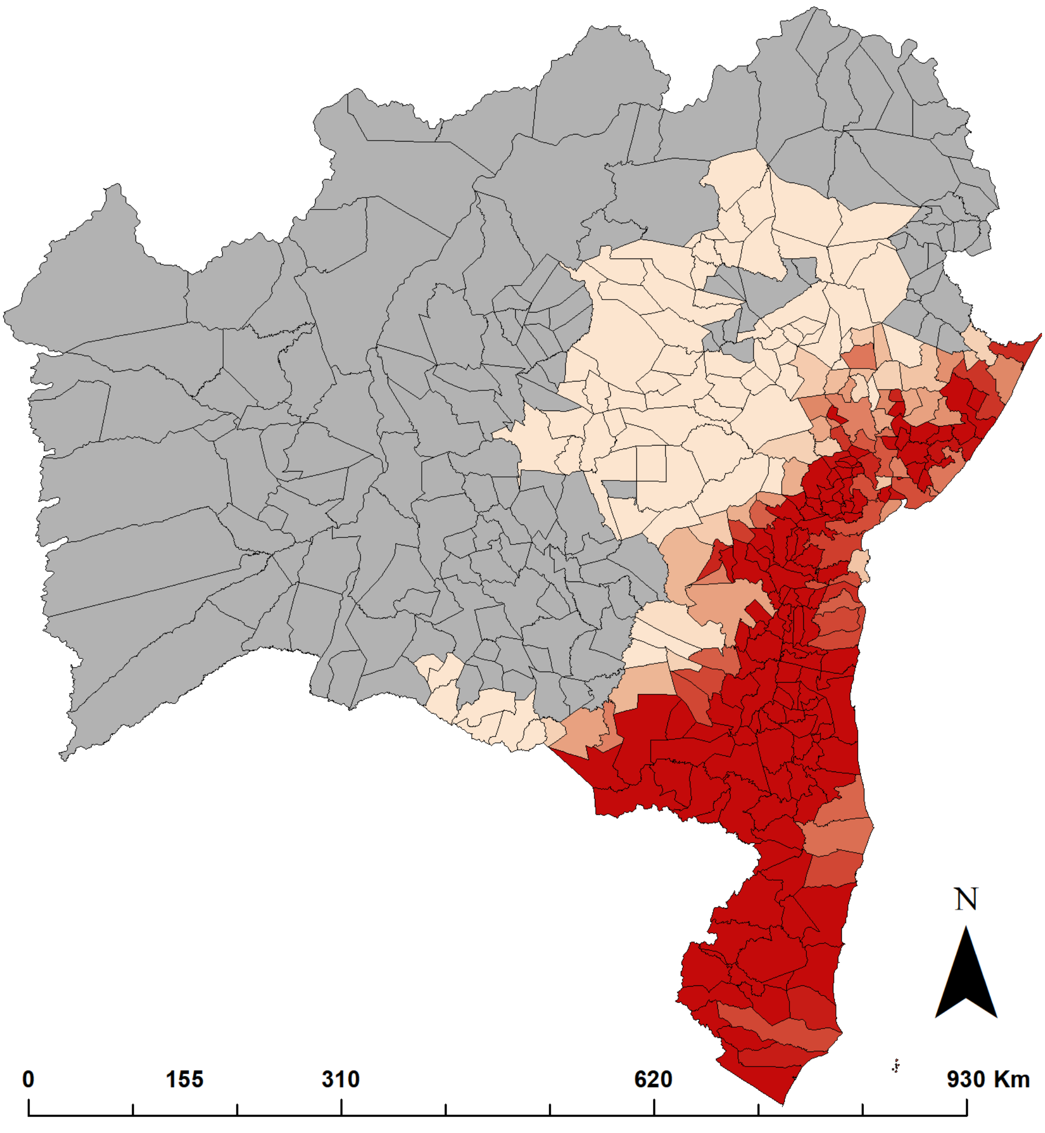

Fig S15. *Panstrongylus geniculatus* ("weighted presence")

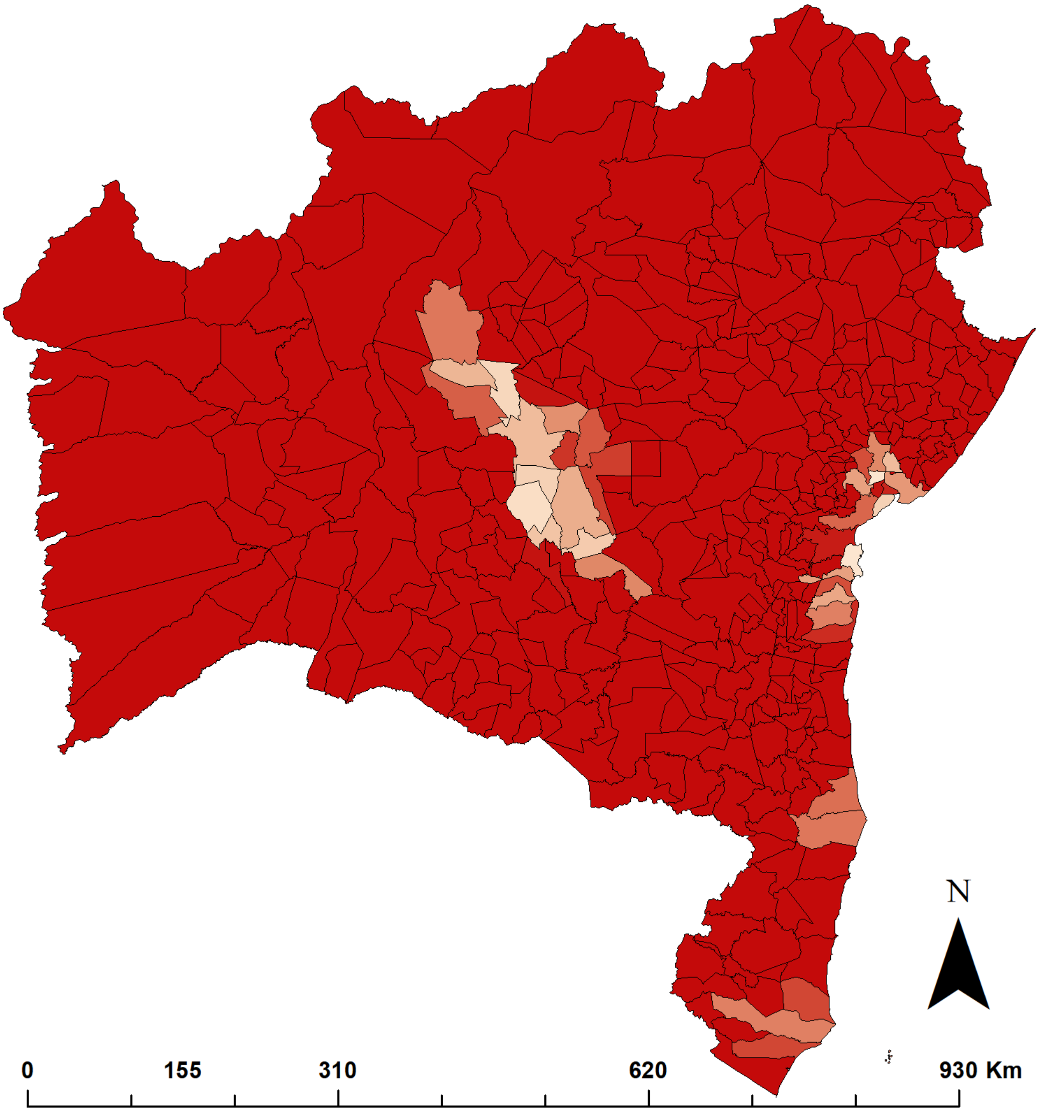

Fig S16. ***Triatoma petrocchiae*** **("weighted presence")**

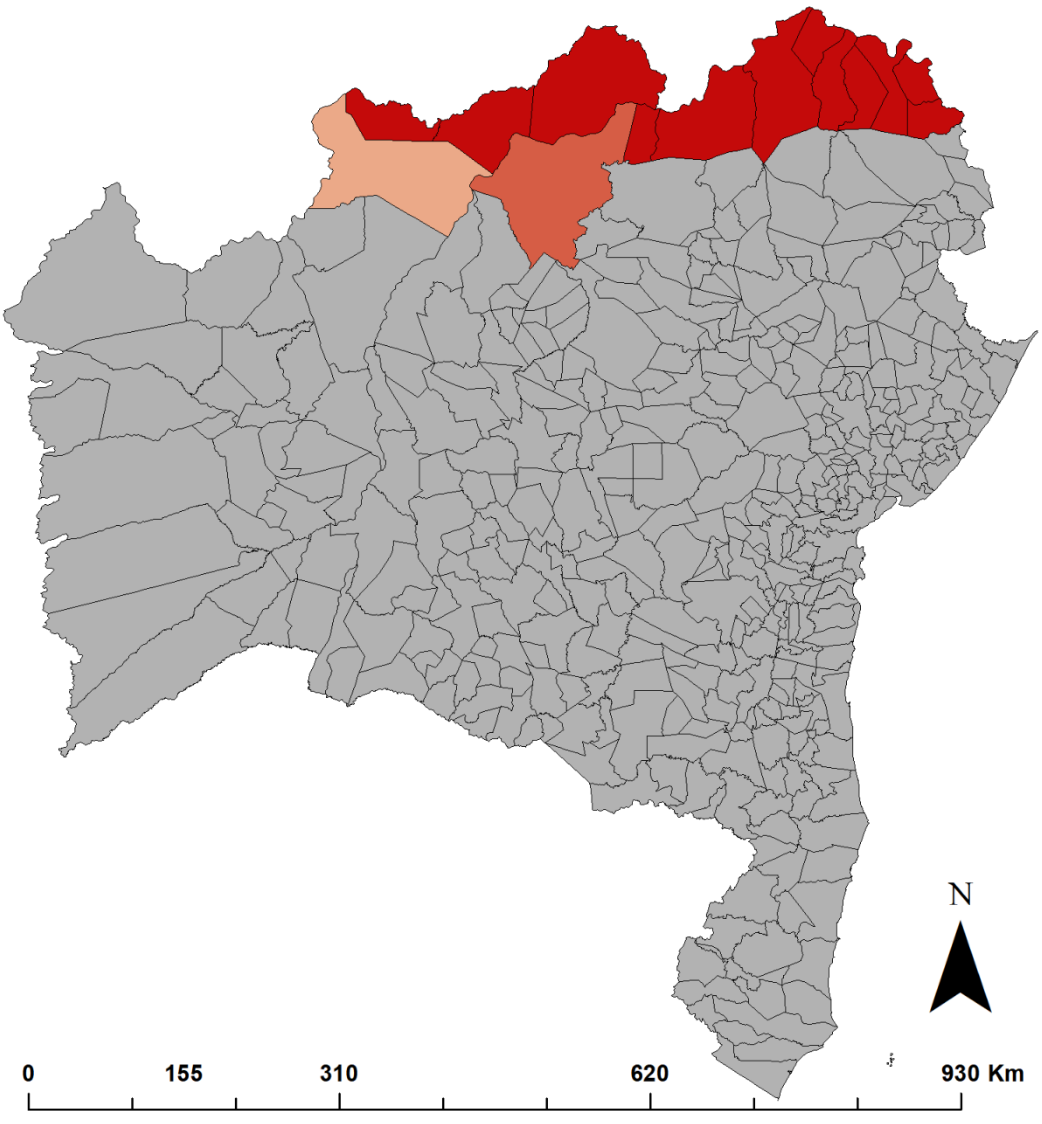

Fig S17. ***Triatoma sherlocki*** **("weighted presence")**

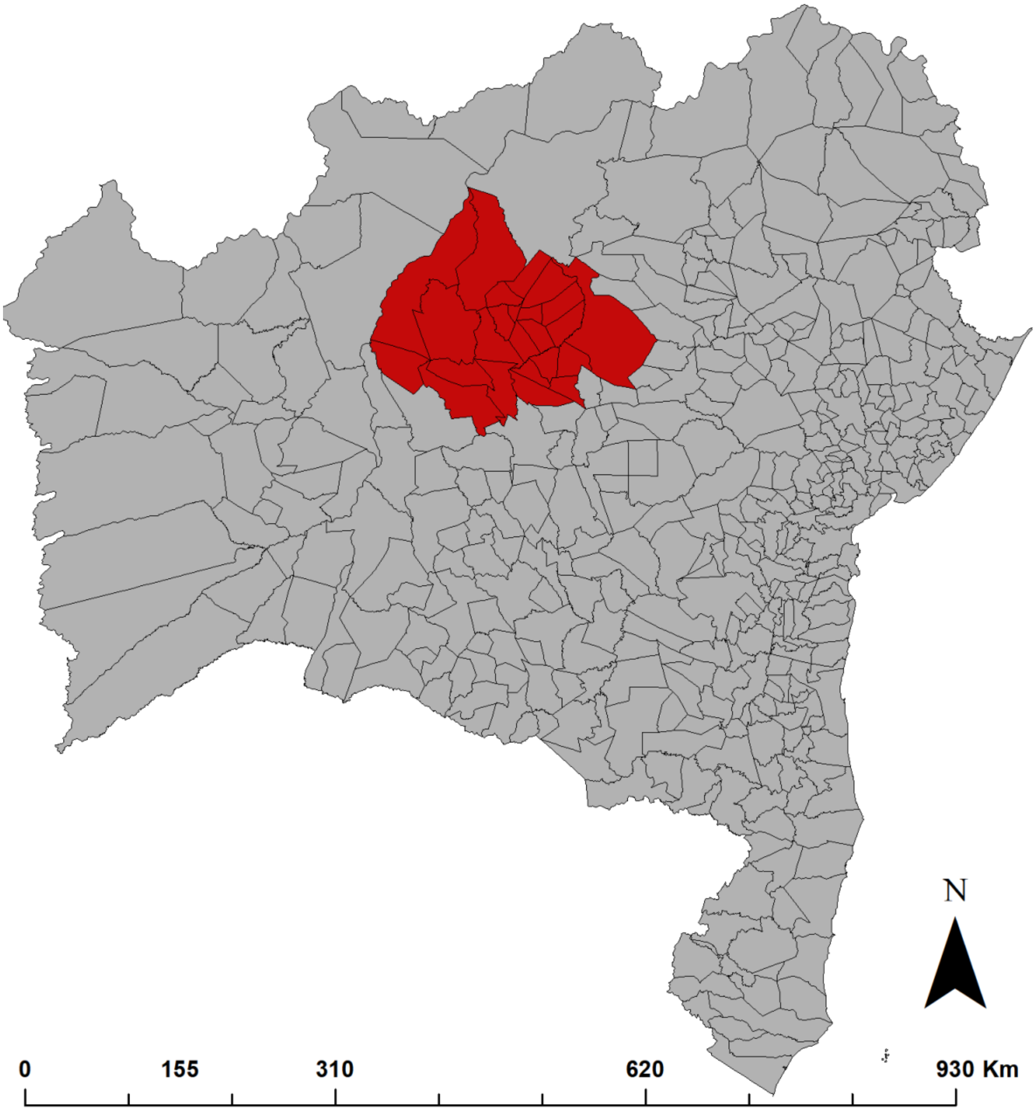

Fig S18. *Triatoma melanocephala* (“weighted presence”)

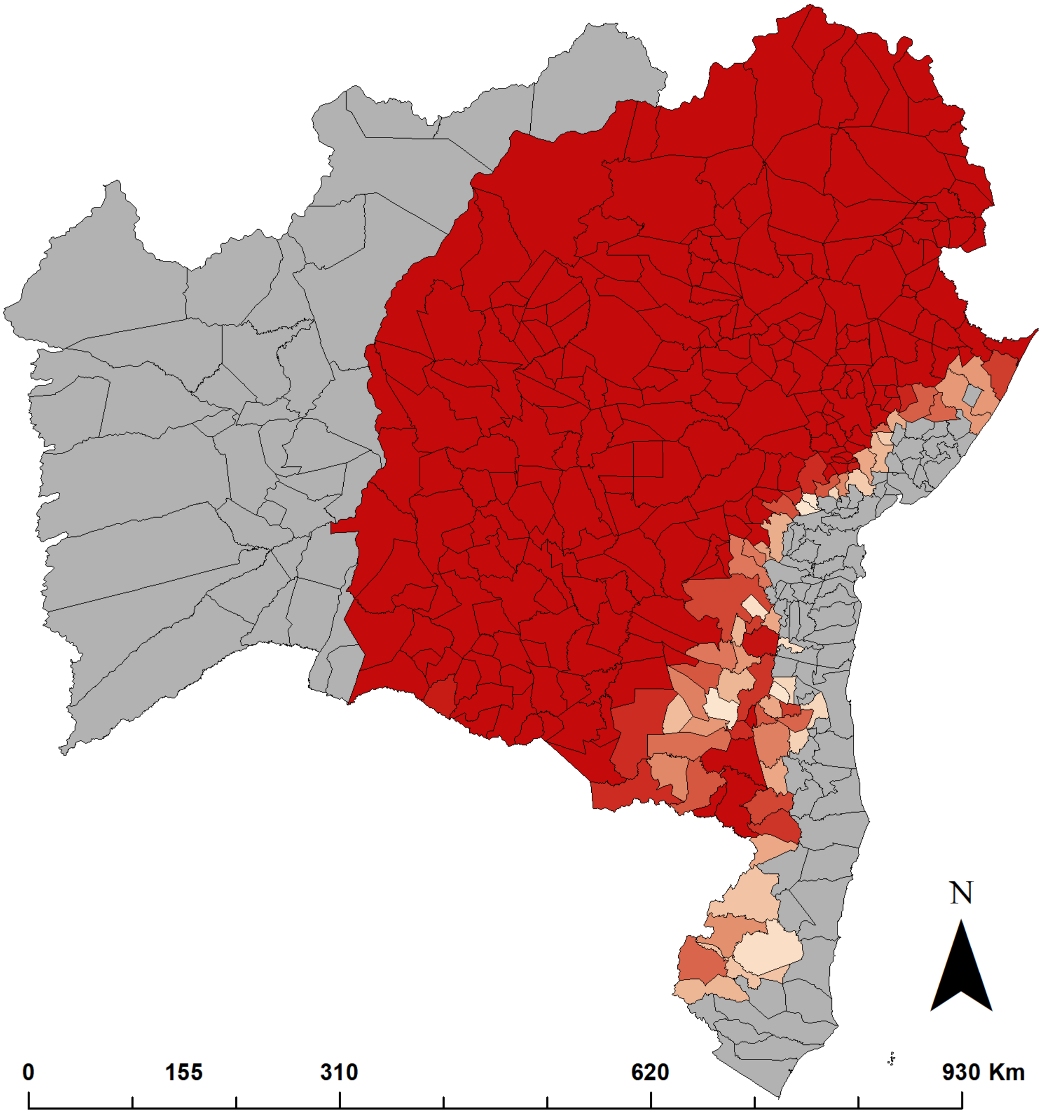

Fig S19. *Panstrongylus diasi* ("weighted presence")

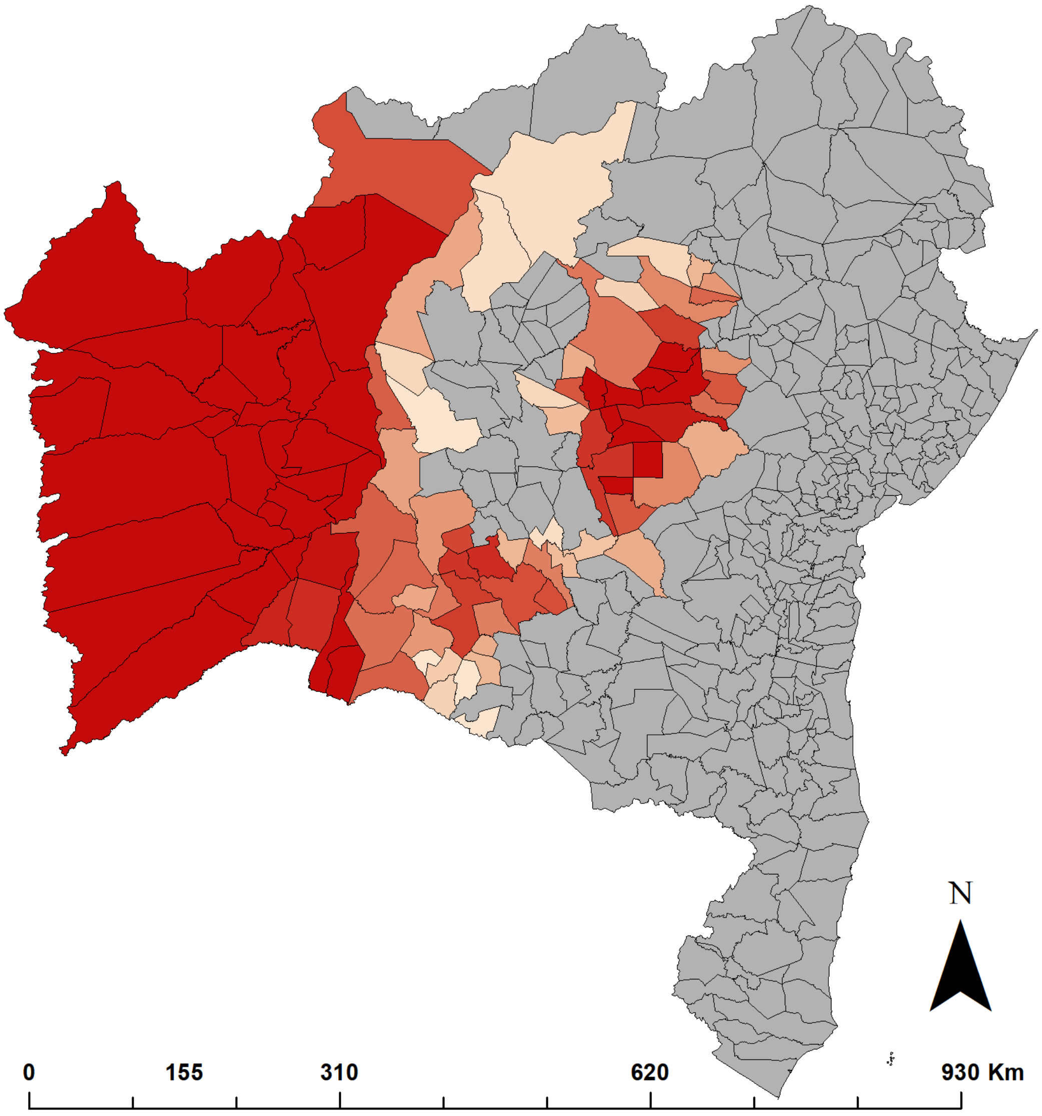

Fig S20. ***Panstrongylus lenti*** **(“weighted presence”)**

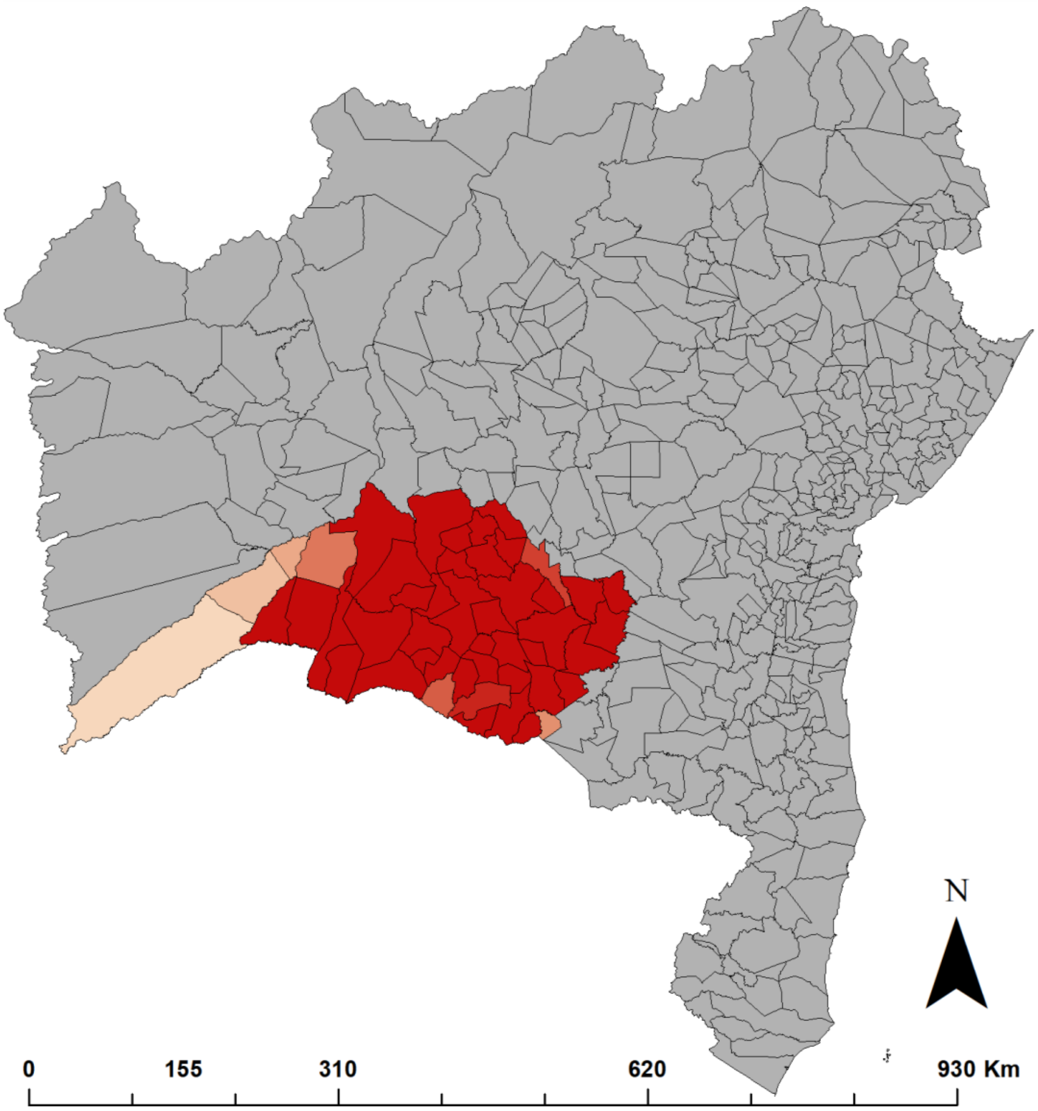

Fig S21. ***Cavernicola pilosa*** **("weighted presence")**

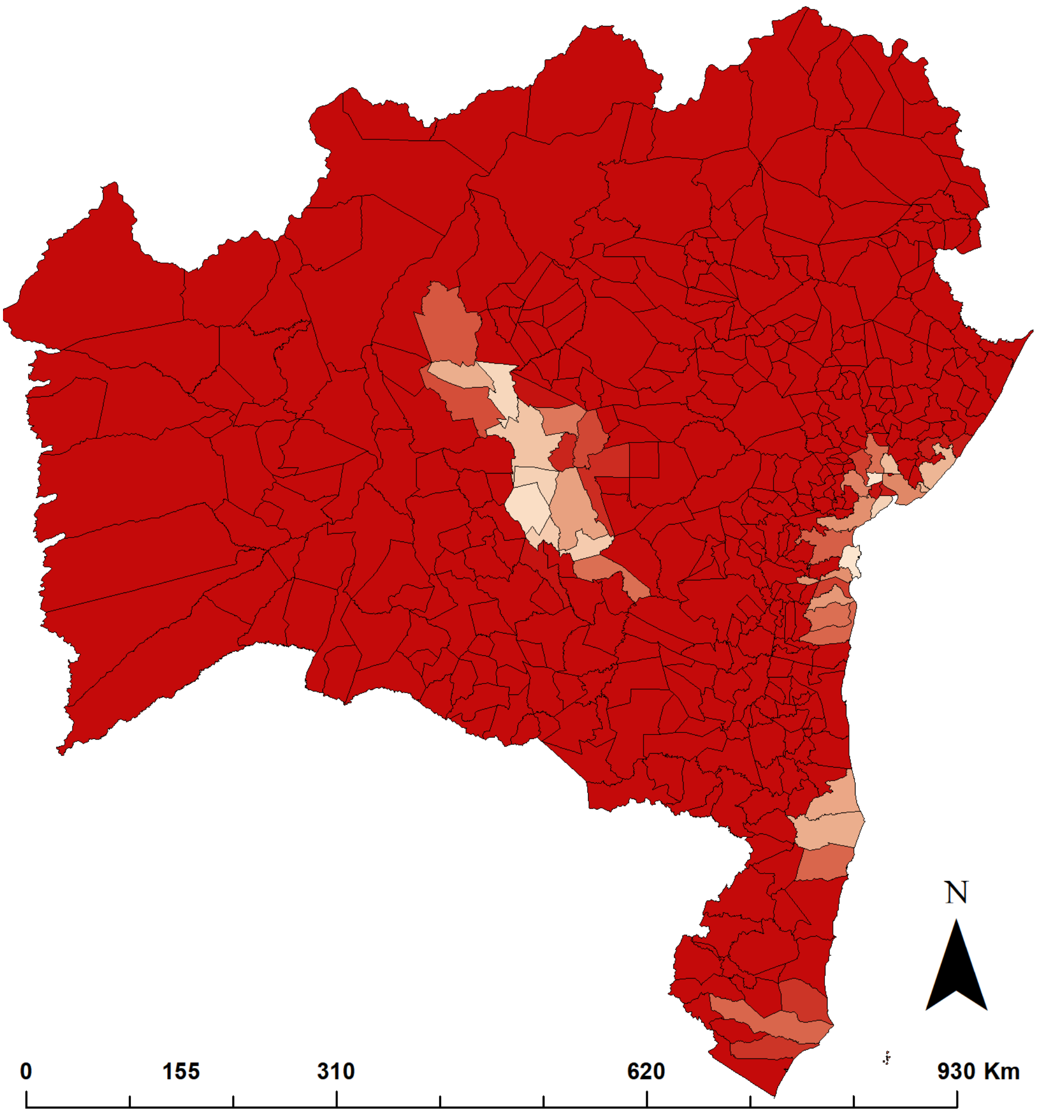

Fig S22. ***Rhodnius domesticus*** **("weighted presence")**

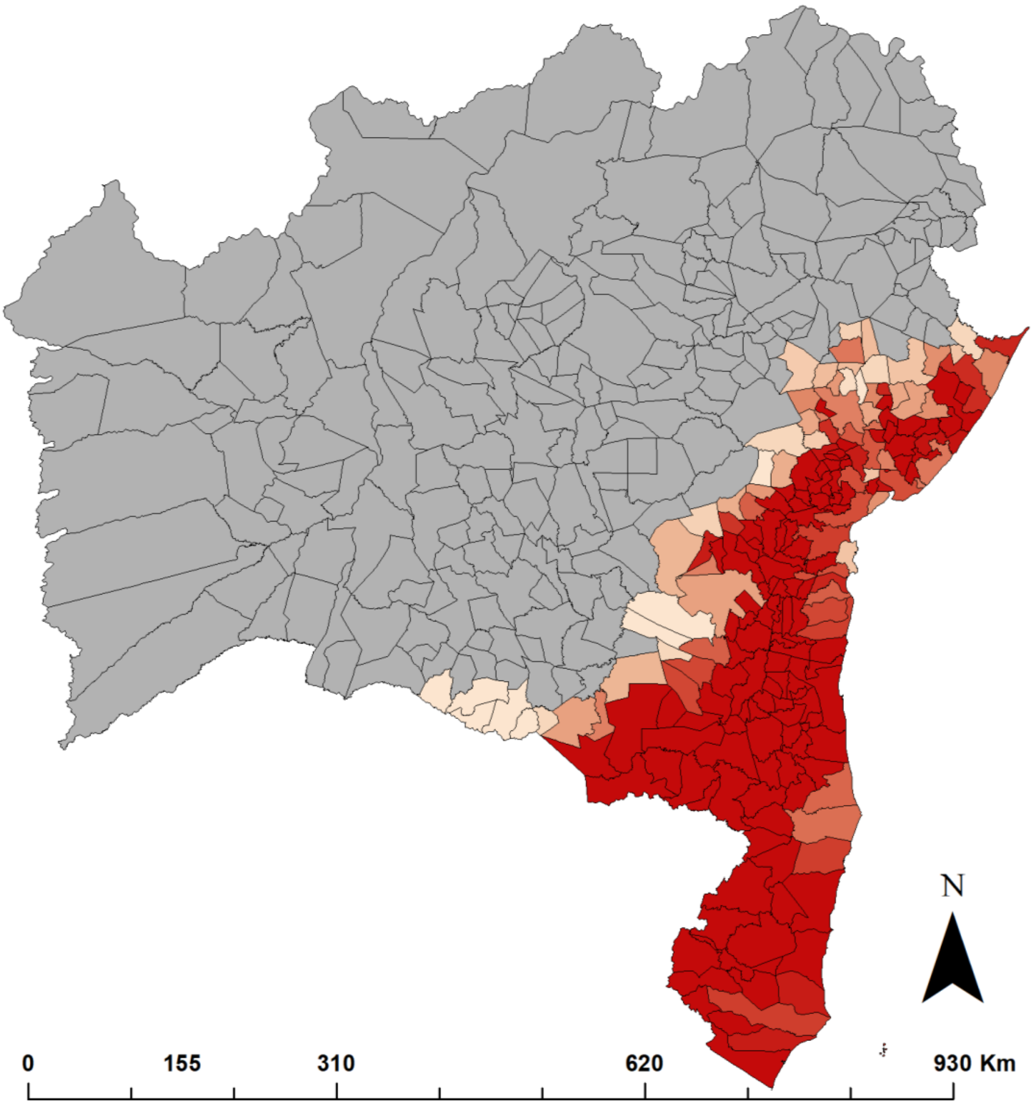

Fig S23. ***Psammolestes tertius*** **("weighted presence")**

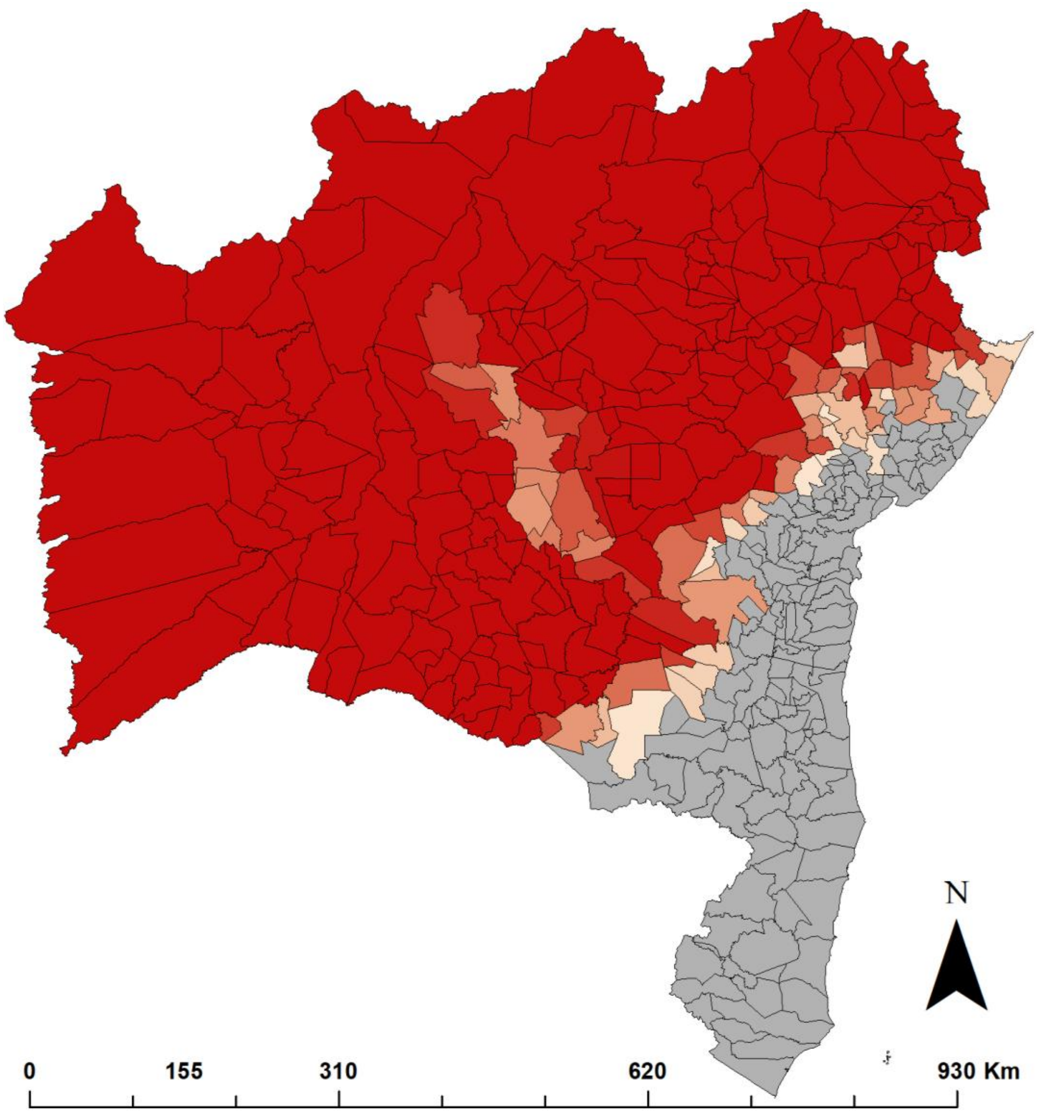

Fig S24. *Parabelminus yurupucu* (“weighted presence”)

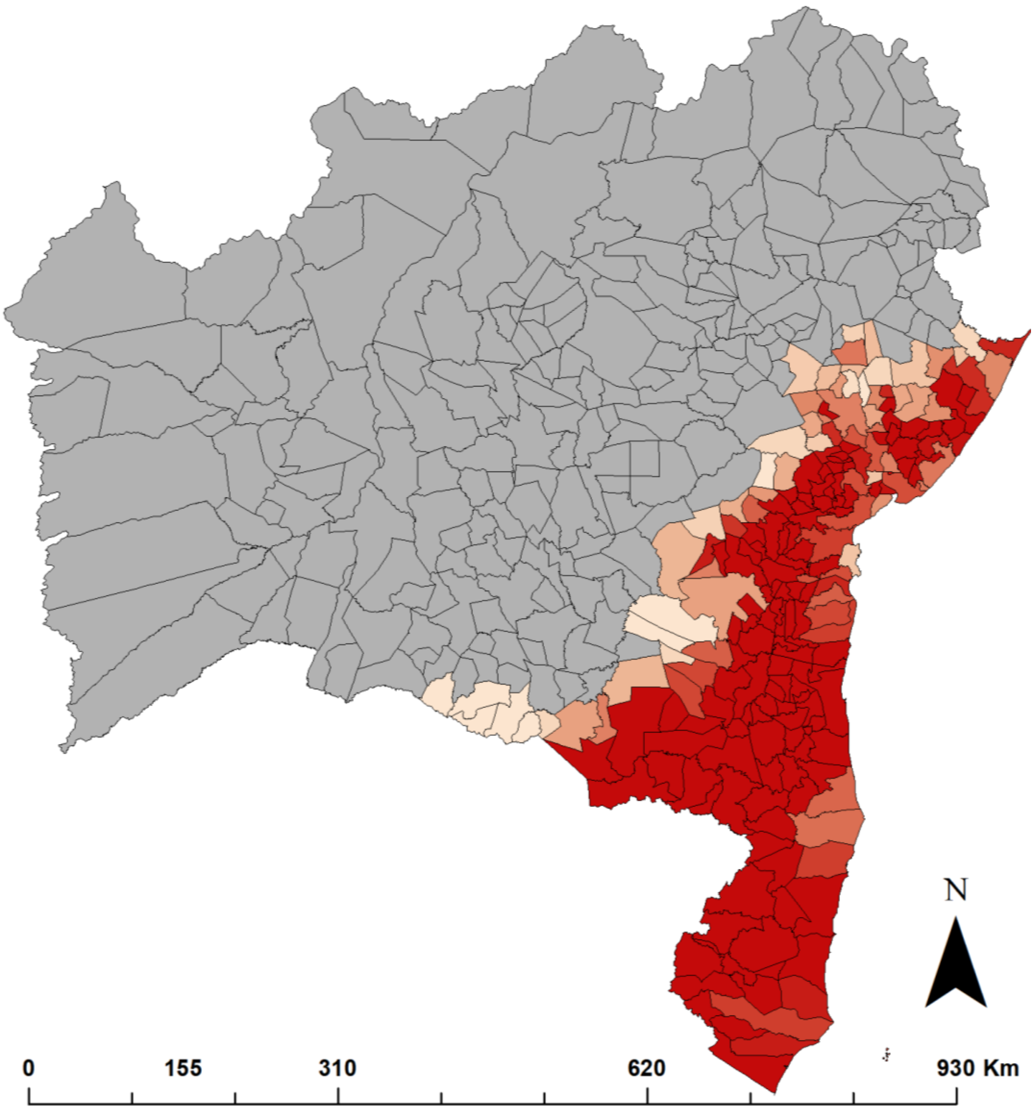

Supplement: Supplementary file 2 — Additional file 2: Figure S1.Triatoma infestans in Bahia, Brazil (since 2006). Figure S2. Triatoma rubrofasciata in Bahia, Brazil (as reported). Figure S3. Triatoma juazeirensis/brasiliensis in Bahia, Brazil (“weighted presence”). Figure S4. Triatoma sordida in Bahia, Brazil (“weighted presence”). Figure S5. Triatoma pseudomaculata in Bahia, Brazil (“weighted presence”). Figure S6. Panstrongylus megistus in Bahia, Brazil (“weighted presence”). Figure S7. Triatoma lenti/bahiensis in Bahia, Brazil (“weighted presence”). Figure S8. Triatoma vitticeps in Bahia, Brazil (“weighted presence”). Figure S9. Triatoma costalimai in Bahia, Brazil (“weighted presence”). Figure S10. Panstrongylus lutzi in Bahia, Brazil (“weighted presence”). Figure S11. Rhodnius neglectus in Bahia, Brazil (“weighted presence”). Figure S12. Rhodnius nasutus in Bahia, Brazil (“weighted presence”). Figure S13. Triatoma melanica in Bahia, Brazil (“weighted presence”). Figure S14. Triatoma tibiamaculata in Bahia, Brazil (“weighted presence”). Figure S15. Panstrongylus geniculatus in Bahia, Brazil (“weighted presence”). Figure S16. Triatoma petrocchiae in Bahia, Brazil (“weighted presence”). Figure S17. Triatoma sherlocki in Bahia, Brazil (“weighted presence”). Figure S18. Triatoma melanocephala in Bahia, Brazil (“weighted presence”). Figure S19. Panstrongylus diasi in Bahia, Brazil (“weighted presence”). Figure S20. Panstrongylus lenti in Bahia, Brazil (“weighted presence”). Figure S21. Cavernicola pilosa in Bahia, Brazil (“weighted presence”). Figure S22. Rhodnius domesticus in Bahia, Brazil (“weighted presence”). Figure S23. Psammolestes tertius in Bahia, Brazil (“weighted presence”). Figure S24. Parabelminus yurupucu in Bahia, Brazil (“weighted presence”). Triatomine bug species occurrence based on routine control-surveillance records (SESAB, 1999–2019) and published reports [17, 27–29, 29–31, 31–34, 40–45]. [file 13071_2021_4954_MOESM2_ESM.pdf]
